# Analysis of Transcriptional Signatures in Response to *Listeria monocytogenes* Infection Reveals Temporal Changes That Result from Type I Interferon Signaling

**DOI:** 10.1371/journal.pone.0150251

**Published:** 2016-02-26

**Authors:** Jonathan M. Pitt, Simon Blankley, Krzysztof Potempa, Christine M. Graham, Lucia Moreira-Teixeira, Finlay W. McNab, Ashleigh Howes, Evangelos Stavropoulos, Virginia Pascual, Jacques Banchereau, Damien Chaussabel, Anne O’Garra

**Affiliations:** 1 Laboratory of Immunoregulation and Infection, The Francis Crick Institute, Mill Hill Laboratory, London, United Kingdom; 2 Baylor Institute for Immunology Research/ANRS Center for Human Vaccines, INSERM, Dallas, Texas, United States of America; 3 The Jackson Laboratory for Genomic Medicine, 263 Farmington Ave, Farmington, CT 06030, Connecticut, United States of America; 4 Systems Immunology, Benaroya Research Institute, Seattle, Washington, United States of America; 5 Sidra Medical and Research Center, Doha, Qatar; 6 Department of Medicine, NHLI, Imperial College, London, United Kingdom; University of Illinois at Chicago College of Medicine, UNITED STATES

## Abstract

Analysis of the mouse transcriptional response to *Listeria monocytogenes* infection reveals that a large set of genes are perturbed in both blood and tissue and that these transcriptional responses are enriched for pathways of the immune response. Further we identified enrichment for both type I and type II interferon (IFN) signaling molecules in the blood and tissues upon infection. Since type I IFN signaling has been reported widely to impair bacterial clearance we examined gene expression from blood and tissues of wild type (WT) and type I IFNαβ receptor-deficient (*Ifnar1*^*-/-*^) mice at the basal level and upon infection with *L*. *monocytogenes*. Measurement of the fold change response upon infection in the absence of type I IFN signaling demonstrated an upregulation of specific genes at day 1 post infection. A less marked reduction of the global gene expression signature in blood or tissues from infected *Ifnar1*^*-/-*^ as compared to WT mice was observed at days 2 and 3 after infection, with marked reduction in key genes such as *Oasg1* and *Stat2*. Moreover, on in depth analysis, changes in gene expression in uninfected mice of key IFN regulatory genes including *Irf9*, *Irf7*, *Stat1* and others were identified, and although induced by an equivalent degree upon infection this resulted in significantly lower final gene expression levels upon infection of *Ifnar1*^*-/-*^ mice. These data highlight how dysregulation of this network in the steady state and temporally upon infection may determine the outcome of this bacterial infection and how basal levels of type I IFN-inducible genes may perturb an optimal host immune response to control intracellular bacterial infections such as *L*. *monocytogenes*.

## Introduction

Infection with the Gram-positive bacterium *Listeria monocytogenes* poses serious risk to immunocompromised individuals, and also in women during pregnancy when the infection can lead to death of the foetus [1, 2].

Murine models of systemic *L*. *monocytogenes* infection are a well-established system to study host immune responses against intracellular pathogenic bacteria, and have provided significant insight and understanding of the mechanisms underlying protection or pathogenesis to intracellular bacteria [1, 3, 4]. The early innate immune response to *L*. *monocytogenes* has been reported to be triggered through several different pathogen recognition pathways, including the Toll-like-receptor (TLR)/MyD88-dependent pathways [5–7], resulting in the production of cytokines, such as interleukin (IL)-12, tumour necrosis factor (TNF) and IL-18 [3]. The cytokines TNF and IL-12 play key roles in host protection against *L*. *monocytogenes* infection [3]. IL-12 drives the successful differentiation of host-protective T helper 1 CD4^+^ T cells (Th1), which produce interferon (IFN)-γ essential for controlling *L*. *monocytogenes* infection [3]. IFN-γ signals through the IFN-γ receptor (IFN-γR) expressed upon infected macrophages to induce IFN-responsive signaling pathways [3], which in turn upregulate macrophage microbicidal processes to destroy and contain the pathogen [8]. In addition to triggering distinct TLR- and NOD-like-receptor (NLR) transcriptional responses [9], *L*. *monocytogenes* can also be recognized within phagosomes [4] and through a STING/IFN regulatory factor (IRF3) cytosolic surveillance pathway results in expression of interferon (IFN)-β [4, 10–12]. The *L*. *monocytogenes* ligand cyclic diadenosine monophosphate is a significant trigger of the cytosolic pathway of innate immunity and is a potent inducer of IFN-β [13]. TRIF mediated type I IFN production of *L*. *monocytogenes* has also been reported [5].

Mice that lack the type I IFNαβ receptor-deficient (*Ifnar1*^*-/-*^) have been shown to be more resistant to infection with *L*. *monocytogenes* compared with wild type (WT) mice [14–16], as have mice deficient for the downstream transcription factor IRF3 [15]. The type I IFN family constitutes several cytokines that each signal through the type I IFNαβ receptor (IFNαβR). Of these, IFN-α (constituting several partially homologous genes) and IFN-β are the best characterized, with IFN-β identified as the major immediate early IFN made during *L*. *monocytogenes* infection [4, 17]. Macrophages and dendritic cells (DC) have been shown to produce the majority of type I IFN following *in vivo* infection with *L*. *monocytogenes* [18, 19], which can act by autocrine or paracrine signaling via the IFNαβR, culminating in the activation of signal transducer and activation of transcription (STAT)-1/STAT-2 intracellular signaling pathways [20]. *L*. *monocytogenes* infection of macrophages has been shown to induce two basic categories of genes: an "early/persistent" cluster consistent with NF-kappaB-dependent responses downstream of TLRs, and a subsequent "late response" cluster largely composed of IFN-responsive genes (IRGs) [21]. Hyper virulence of certain *Listeria* species observed after infection in mice correlates with heightened type I IFN production *in vivo* [22]. Mechanisms identified for how type I IFN impairs the protective immune response to *L*. *monocytogenes* include the apoptosis of T cells [15, 23], reduced number and effector function of TNF producing innate immune cells [14], induction of IL-10 by phagocytic cells following type I IFN-induced apoptosis [24] and down-regulation of the IFN-γR [25] impeding the signaling required to eradicate the pathogen.

Microarray analysis of macrophages infected with *L*. *monocytogenes* has previously revealed a strong upregulation of IFN regulated gene clusters controlled by both TLR and cytosolic signaling in these cells [21]. However how perturbation of type I IFN signaling in the whole organism affects transcriptional response in *L*. *monocytogenes* infected mice has not been reported. Transcriptional microarray approaches to study host gene expression signatures in the whole organism in response to infection or vaccination have yielded a wealth of information on how various factors and molecular pathways can lead to pathogenesis [26], or to protective immunity [27–29]. Thus the use of transcriptional analysis is well placed to help extricate some of the complexities of type I IFN signaling during *L*. *monocytogenes* infection.

## Results

### Type I and type II IFN responsive genes are associated with *L*. *monocytogenes* infection *in vivo* in both blood and spleen

**Fig 1:**
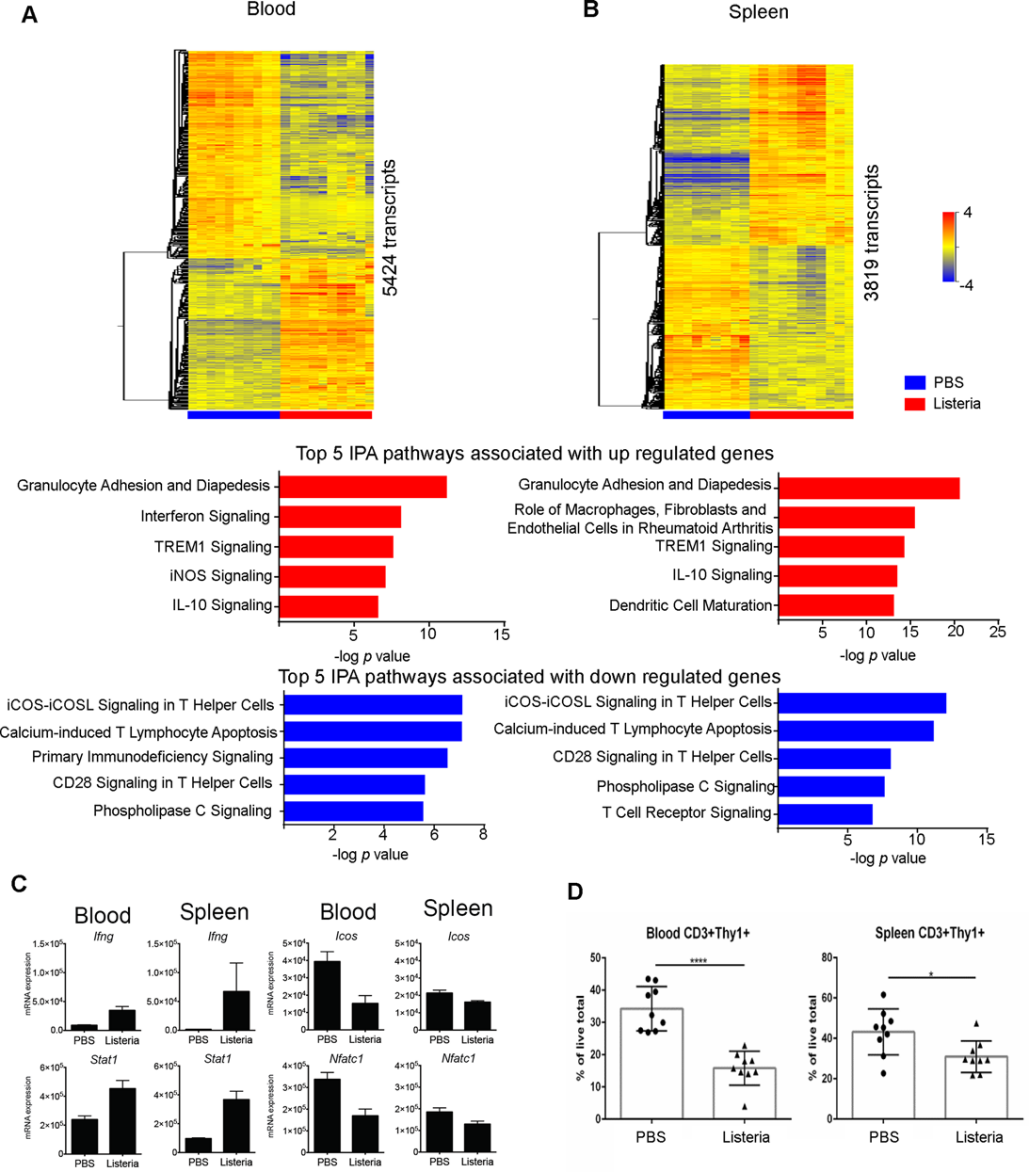
Transcriptional response following *L*. *monocytogenes* infection of WT mice in blood and spleen. **(A, B)** Heatmaps of the significantly regulated blood and spleen transcripts at day 3 following intravenous injection of C57BL/6 WT mice with 5 × 10^3^ of *L*. *monocytogenes* (*p* < 0.01 after unpaired *t*-test with Benjamini–Hochberg multiple testing correction on transcripts passing quality control filtering, *n* = 10 mice/group). The top five QIAGEN Ingenuity® Pathway Analysis (IPA®) canonical pathways by significance (Fisher’s exact test) for upregulated (red bars) or downregulated (blue bars) genes are listed below the heatmaps. **(C)** qRT-PCR data on selected transcripts normalized relative to *Hprt1* expression levels (mean with SD, *n* = 5 mice/group). Samples are from the same experiment as the microarray data. **(D)** CD3^+^Thy1^+^ cells in blood and spleen as a percentage of total live cells. Pooled results are from triplicates of 3 independent experiments (mean with SEM, *n* = 3 mice/group/experiment).

In order to elucidate the host transcriptional response following *L*. *monocytogenes* infection, C57BL/6 mice were infected with *L*. *monocytogenes* or administered volume-matched PBS as a control (hereafter referred to as uninfected). RNA was obtained from blood and spleen of infected and uninfected mice at day 3 post infection and processed for microarray. At day 3 following infection a mean of 10^8^ colony forming units (CFU) of *L*. *monocytogenes* was detected in the spleen (S1 Fig). Unbiased principle component analysis (PCA) on those transcripts that were significantly detected above background expression showed a clear separation of *L*. *monocytogenes* infected animals from the uninfected controls, in both the blood (19965 transcripts) and the spleen (20914 transcripts) (S1 Fig). Following statistical filtering (Unpaired t-test with Benjamini Hochberg multiple testing correction, *P* < 0.01), 5424 and 3819 significantly differentially expressed transcripts were identified from the blood and spleen respectively (Fig 1A and 1B). The number of transcripts that were either up or down regulated following *L*. *monocytogenes* infection was calculated (S1 Fig) and IPA canonical pathway analysis undertaken on these separated up and down regulated (compared to uninfected controls) genes (Fig 1A and 1B).

Following infection in both blood and spleen there was upregulation of canonical pathways including “Granulocyte Adhesion and Diapedesis”, “TREM1 Signaling” and “IL-10 Signaling”. The top downregulated pathways in both the blood and the spleen involved lymphocyte signaling pathways and T lymphocyte apoptosis pathways (Fig 1A and 1B) that included lower abundance of several genes following *L*. *monocytogenes* infection such as *Cd4*, *Cd28*, *Icos*, *Nfatc1*, *Nfatc2* and *Trat1*. Such changes in gene expression were validated by RT-PCR for the genes *Icos* and *Nfatc1* (Fig 1C) and also validated by microarray analysis of blood and spleen from an independent infection experiment (data not shown). These observations correlated with the reduction in the percentage of CD3^+^Thy1^+^ cells in the blood and spleen following infection as shown by flow cytometry analysis of infected compared to uninfected mice (Fig 1D, S2 Fig).

**Fig 2:**
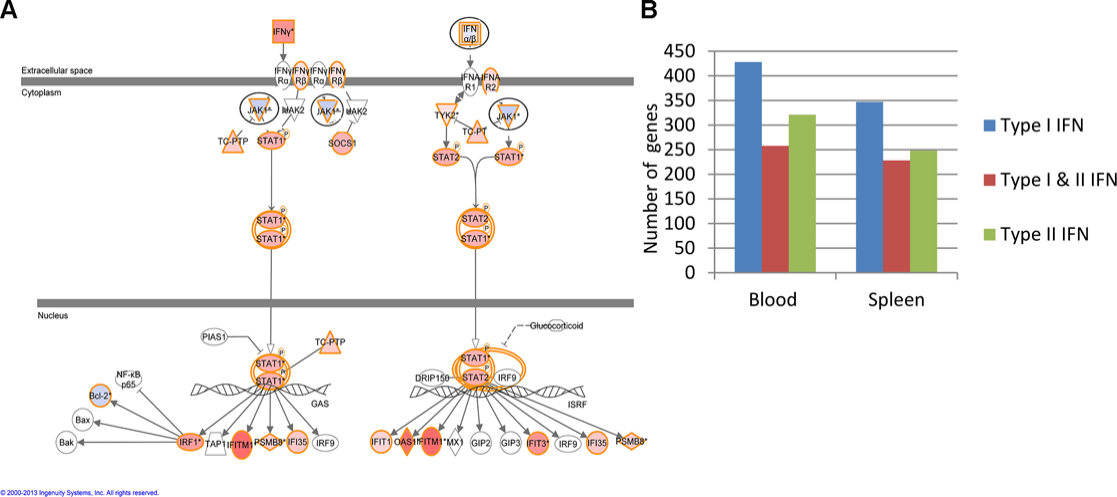
*L*. *monocytogenes* infection modulates a number of IFN signaling pathway and IFN response genes in the blood and spleen. **(A)** The IFN signaling pathway (QIAGEN Ingenuity® Pathway Analysis) was overlaid with the day 3 post infection blood genes shown in Fig 1A. Genes (IFNαβ and JAK1) with opposite expression patterns between blood and spleen are highlighted with black circles. Red: upregulated, Blue: downregulated. **(B)** IFN response genes (type I, type II, and type I and II) associated with blood and spleen transcripts reported in Fig 1A and 1B and the Interferome database (www.interferome.org) were quantitated.

In addition the IPA canonical pathway “Interferon signaling” was found to be significantly enriched in both blood and spleen analyses (-log *P* values 8.0 and 6.2 respectively). We identified IFN-γ as the predicted most activated upstream molecule in the blood and spleen following *L*. *monocytogenes* infection (data not shown). Transposition of gene expression data onto the IFN canonical pathway illustrated many significantly differentially regulated genes following *L*. *monocytogenes* infection (Fig 2A). Focusing on this signaling pathway revealed that the significantly differentially expressed genes within this pathway were broadly similar between blood and spleen including *Ifng*, *Ifngr2*, *Tc-ptp*, *Stat1*, *Stat2*, *Socs1*, *Ifi35*, *Ifit3*, *Ifitm1*, *Oas1*, *Ifit1*, *Psmb8*, *Tap1*, *Irf1*, *Bak*, *Bax* and *Bcl2* (Fig 2A). Such changes in gene expression were validated by RT-PCR for the genes *Ifng* and *Stat1* (Fig 1C) and also validated by microarray analysis of blood and spleen from an independent infection experiment (data not shown). In contrast *Ifnar2* and *Jak1* were identified in the blood but not the spleen, “IFNαβ”, was down regulated following *L*. *monocytogenes* infection from blood (gene–*Ifna4*) and upregulated in the spleen (gene–*Ifnb1*). Using the Interferome v2 database [30] we identified from the significantly expressed transcript lists in both blood and spleen a set of genes classified as being type I, type I & II or type II IFN responsive (Fig 2B). However in both blood and spleen there were more genes associated with type I IRGs than type II IRGs.

### The transcriptional fold change varies differentially at different time points after *L*. *monocytogenes* infection in WT and *Ifnar1*^*-/-*^ mice

**Fig 3:**
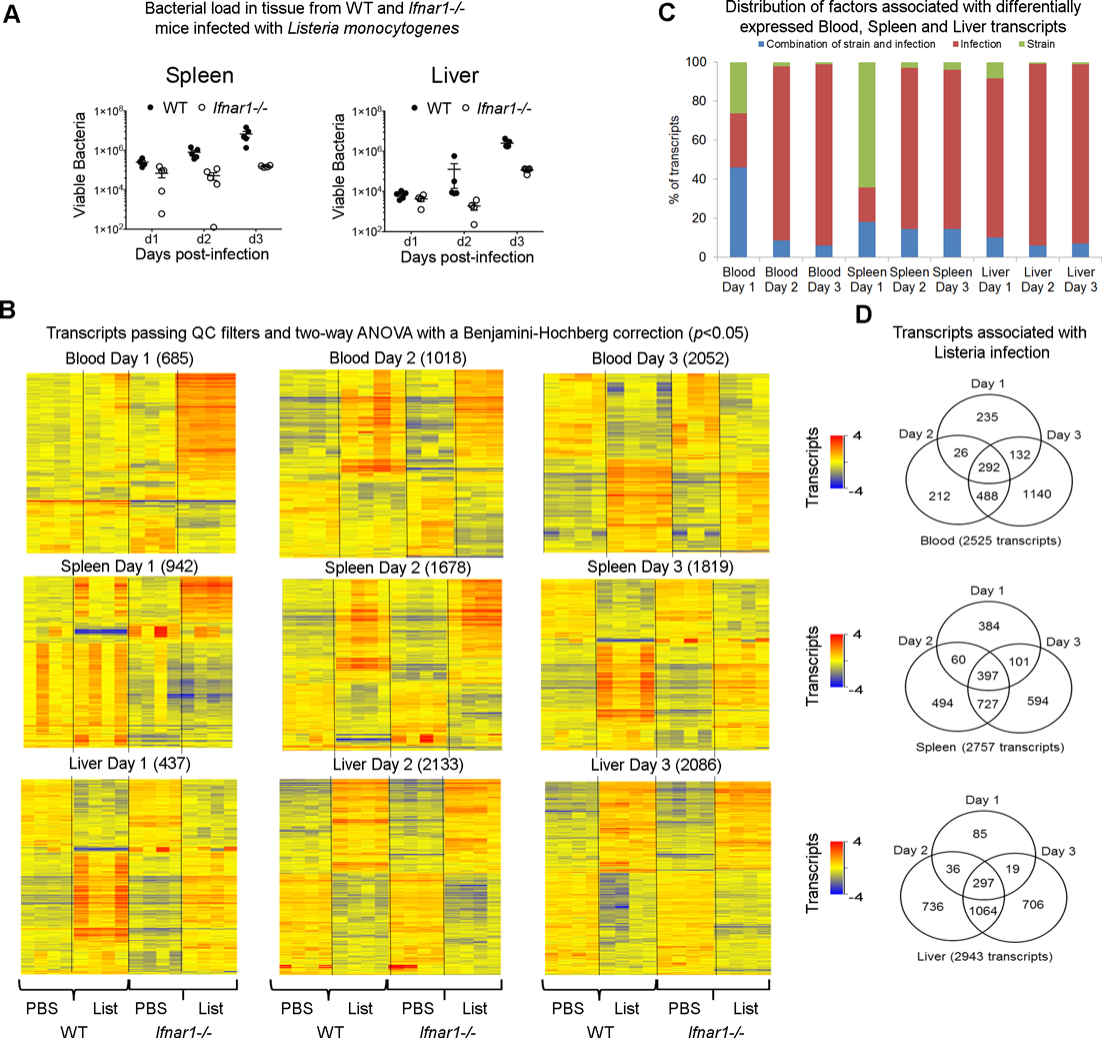
Tissue-specific transcriptional responses of WT and *Ifnar1*^-/-^ mice in blood, spleen and liver following *L*. *monocytogenes* infection at different time points and bacterial loads. **(A)** Colony forming unit (CFU) values were assessed from the spleen and liver of C57BL/6 WT and *Ifnar1*^**-/-**^ mice at day 1, day 2 and day 3 following intravenous injection with 5 × 10^3^ of *L*. *monocytogenes* (mean with SEM, *n* = 4 or 5 mice/group). **(B)** Heatmap representations of differentially expressed transcripts at different times (days 1, 2 and 3) in blood, spleen, liver from WT and *Ifnar1*^**-/-**^ infected mice relative to uninfected controls (*p* < 0.05 after 2-way ANOVA with Benjamini–Hochberg multiple testing correction on transcripts passing quality control filtering, *n* = 3 or 4 mice/group). **(C)** Percentage of transcripts that were significant for infection alone, strain alone or involving a combination of strain and infection across the different tissues and the infection time course. **(D)** Venn diagrams showing temporal differences and similarities in gene expression in blood, spleen and liver after infection.

Since type I IFN contributes to pathogenesis of intracellular bacterial infection [14–16] and type I IFN-inducible genes were highly represented in both the blood and tissue transcriptional signature during *L*. *monocytogenes* infection we sought to determine how a lack of type I IFN receptor signaling in *Ifnar1*^*-/-*^ mice affected the global transcriptional response to infection as compared to that observed in the WT controls (C57BL/6 background). Following *L*. *monocytogenes* infection the bacterial load (CFU) in the spleen and liver of the mice was significantly lower in the *Ifnar1*^*−/−*^ mice as compared to WT mice at day 3 post infection (Fig 3A), as has been shown previously [14–16]. However, at day 1 post infection little to no change in bacterial load was observed and only a slight reduction was observed at day 2 post infection in the *Ifnar1*^*−/−*^ as compared to WT mice. Unbiased PCA of transcripts significantly detected from background expression could clearly separate *Ifnar1*^*-/-*^ and WT *L*. *monocytogenes* infected mice from uninfected controls in blood, spleen and liver (S3 Fig). There was a degree of separation observed between WT and *Ifnar1*^*-/-*^ mouse strains in either infected or uninfected mice, although this was to a lesser extent (S3 Fig). Statistical filtering (2-way ANOVA with Benjamini Hochberg multiple testing correction, *P* <0.05) revealed 685, 942 and 437 significantly differentially expressed transcripts in blood, spleen and liver at day 1 post infection which were differentially expressed in infected and uninfected *Ifnar1*^*-/-*^ or WT mice (Fig 3B). Strikingly, an upregulation of a significant number of transcripts was observed at day 1 post infection in blood of the *Ifnar1*^*-/-*^ as compared to WT mice (Fig 3B and 3D), although there was not a significant change in the bacterial load at this time post infection. Greater differences in gene expression as a result of *L*. *monocytogenes* infection were observed in both WT and *Ifnar1*^*-/-*^ as compared to uninfected mice by day 2 post infection, and this increased at day 3 post infection (Fig 3B–3D). For all three tissues the majority of transcripts identified as significantly differentially regulated were as a result of infection alone at days 2 and 3 post infection, with small differences due to strain (i.e. WT versus *Ifnar1*^*-/-*^ mice, uninfected) or strain plus infection (i.e. WT versus *Ifnar1*^*-/-*^ mice, infected) (Fig 3C). In contrast at day 1 post-infection there were major changes in gene expression in blood and spleen observed as a result of strain plus infection (i.e. WT versus *Ifnar1*^*-/-*^ mice, infected) (Fig 3C). A change in transcript expression was identified as significantly different due to strain alone (i.e. WT versus *Ifnar1*^*−/−*^ mice) irrespective of infection and is discussed in more detail later. It should be noted that although there was perturbation of a larger number of transcripts at days 2 and 3 as a result of infection, in blood and tissues of both *Ifnar1*^*-/-*^ and WT mice, unique transcripts were also perturbed at each of the different time points post-infection in *Ifnar1*^*-/-*^ and WT mice, with some differentially expressed common transcripts observed between time points (Fig 3D). This demonstrates the dynamic changes that occur temporally in *Ifnar1*^*-/-*^ and WT mice during *L*. *monocytogenes* infection.

### Canonical pathways associated with blood transcripts are differentially expressed in WT versus *Ifnar1*^−/−^ mice infected with *L*. *monocytogenes*

**Fig 4:**
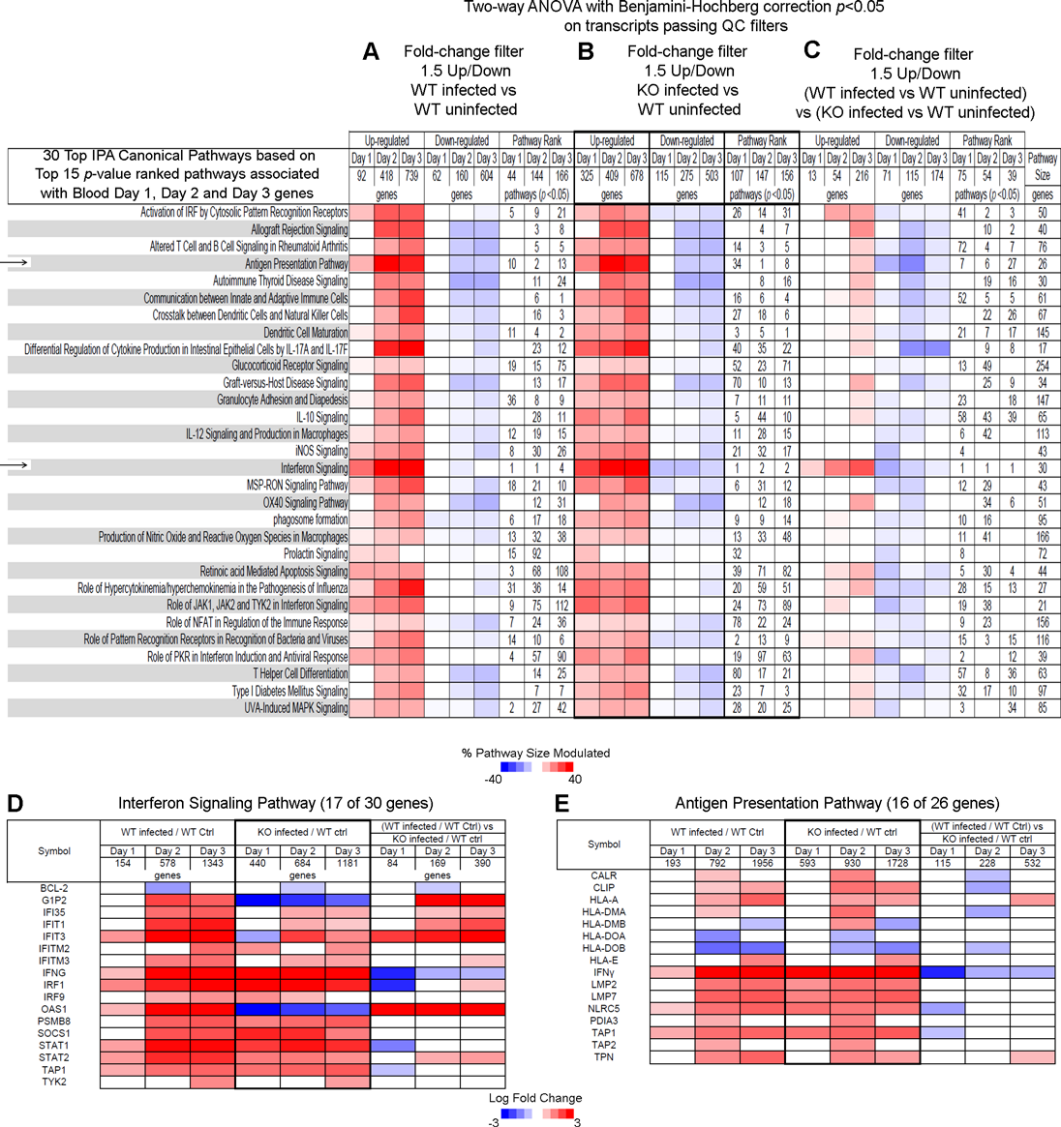
Canonical pathways associated with blood transcripts that are differentially expressed in *L*. *monocytogenes* infected WT versus *Ifnar1*^-/-^ mice against control uninfected WT mice. Top IPA® canonical pathways that are associated with differentially expressed blood day 1, day 2 and day 3 transcripts shown in Fig 3B and that pass a further 1.5-fold change filter ratio between **(A)** WT infected to WT uninfected; **(B)** KO infected to WT uninfected; and **(C)** (WT infected to WT uninfected) as compared to (KO infected to WT uninfected). Percent pathway modulation relative to each dataset and pathway size is indicated in red for up-regulated and blue for down-regulated genes. Pathway rank for pathways passing *p<0*.*05* after Fisher’s Exact test at each time-point is marked. **(D and E)** Detailed heat map of differentially expressed genes found in the **(D)** Interferon Signaling Pathway; and **(E)** Antigen Presentation Pathway.

**Fig 5:**
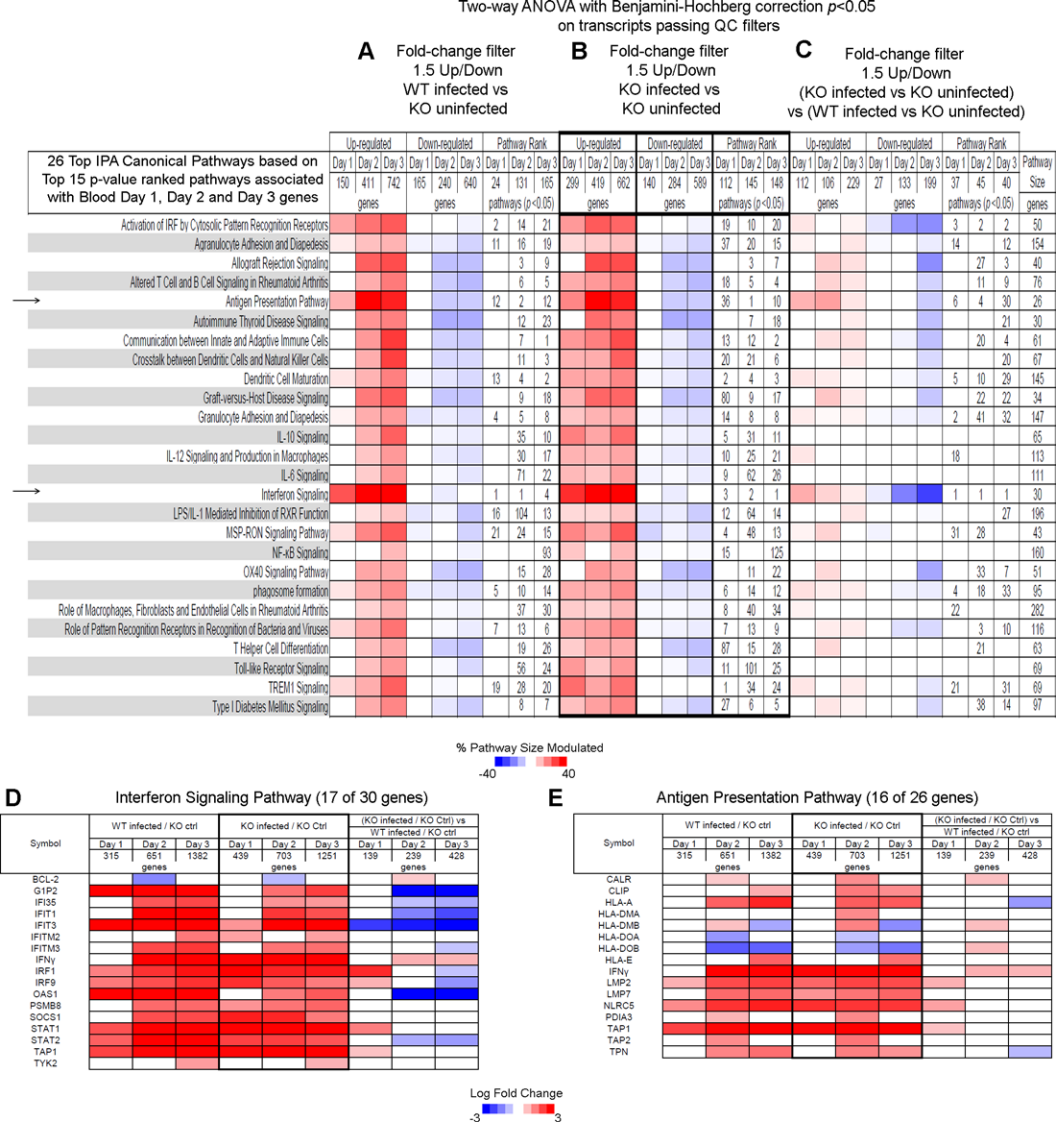
Canonical pathways associated with blood transcripts that are differentially expressed in *L*. *monocytogenes* infected *Ifnar1*^-/-^ versus WT mice against control uninfected *Ifnar1*^-/-^ mice. Top IPA® canonical pathways that are associated with differentially expressed blood day 1, day 2 and day 3 transcripts from Fig 3B and pass a further 1.5-fold change filter ratio between **(A)** WT infected to KO uninfected; **(B)** KO infected to KO uninfected; and **(C)** (KO infected to KO uninfected) as compared to (WT infected to KO uninfected). Percent pathway modulation relative to each dataset and pathway size is indicated in red for up-regulated and blue for down-regulated genes. Pathway rank for pathways passing *p<0*.*05* after Fisher’s Exact test at each time-point is marked. **(D and E)** Detailed heat map of differentially expressed genes found in the **(D)** Interferon Signaling Pathway; and **(E)** Antigen Presentation Pathway.

To better understand how type I IFN signaling may negatively regulate bacterial clearance during *L*. *monocytogenes* infection we set out to identify those transcripts, which temporally differed between WT and *Ifnar1*^*-/-*^ at days 1, 2 and 3 post infection, using a two-step filtering process. From the two-way ANOVA lists generated in Fig 3B, S2 Table, blood transcripts which were inducible in the WT or the *Ifnar1*^*-/-*^ mice by at least 1.5FC upon infection were identified (Figs 4A and 5B) for each time point post infection. Since significant numbers of transcripts were perturbed at each time point post infection the datasets were analyzed using Ingenuity Pathway Analysis (IPA) and the top 15 *p-value* ranked pathways were first identified in infected versus uninfected WT mice at each time point, yielding a total top 30 canonical pathways unique or shared within each of the three time points (Fig 4A). The same fold change followed by IPA analysis was also applied to blood transcripts from the *Ifnar1*^*-/-*^ upon infection as compared to uninfected WT and the 30 canonical pathways defined from Fig 4A were applied to the data and ranked as shown (Fig 4B). These transcripts were then further filtered to retain those which were at least 1.5FC different in blood from infected WT as compared to infected *Ifnar1*^*-/-*^ mice, all relative to blood from control uninfected mice and these transcripts were then subjected to IPA analysis (Fig 4C). This identified the final net difference in the maximal gene expression change upon infection in WT as compared to the *Ifnar1*^*-/-*^, having taken into account the WT baseline difference.

Distinct percentages of up and down modulated genes were observed in those pathways at each time point post infection (Fig 4A–4C). A substantial set of the top pathways was common and similarly perturbed in the blood of both WT and *Ifnar1*^*-/-*^ mice at each time point post infection (Fig 4A and 4B) in keeping with the global transcript analysis observed in Fig 3B and 3C. However, further in depth analysis of transcripts that were perturbed by at least 1.5FC in the blood of infected WT as compared to infected *Ifnar1*^*-/-*^ mice (Fig 4C) revealed that some of these top canonical pathways were differently modulated in the absence of type I IFN signaling at different stages after infection. The IFN signaling pathway ranked as the top canonical pathway for all time points post infection, found to be differentially perturbed in the blood of infected WT as compared to infected *Ifnar1*^*-/-*^ mice (Fig 4C). A higher percentage of down-modulated genes was observed in the blood of infected WT mice as compared to the infected *Ifnar1*^*-/-*^ mice at day 1 post infection (when the bacterial load was identical) (Fig 4C). In contrast, a higher percentage of up-modulated genes was observed in the blood of infected WT mice as compared to the infected *Ifnar1*^*-/-*^ mice at days 2 and 3 post infection (where the bacterial load was reduced in the *Ifnar1*^*-/-*^ mice; (Fig 3A)) (Fig 4C). This demonstrates that type I IFN has different roles in gene regulation at different stages after infection. Other pathways with a higher percentage of down-modulated genes at day 1 and up-modulated genes at day 2 and 3, or day 3 only, post infection in WT compared to *Ifnar1*^*-/-*^ mice included activation of IRF by cytosolic pattern recognition receptors and the antigen presenting and dendritic cell maturation pathways respectively (Fig 4C).

**Fig 6:**
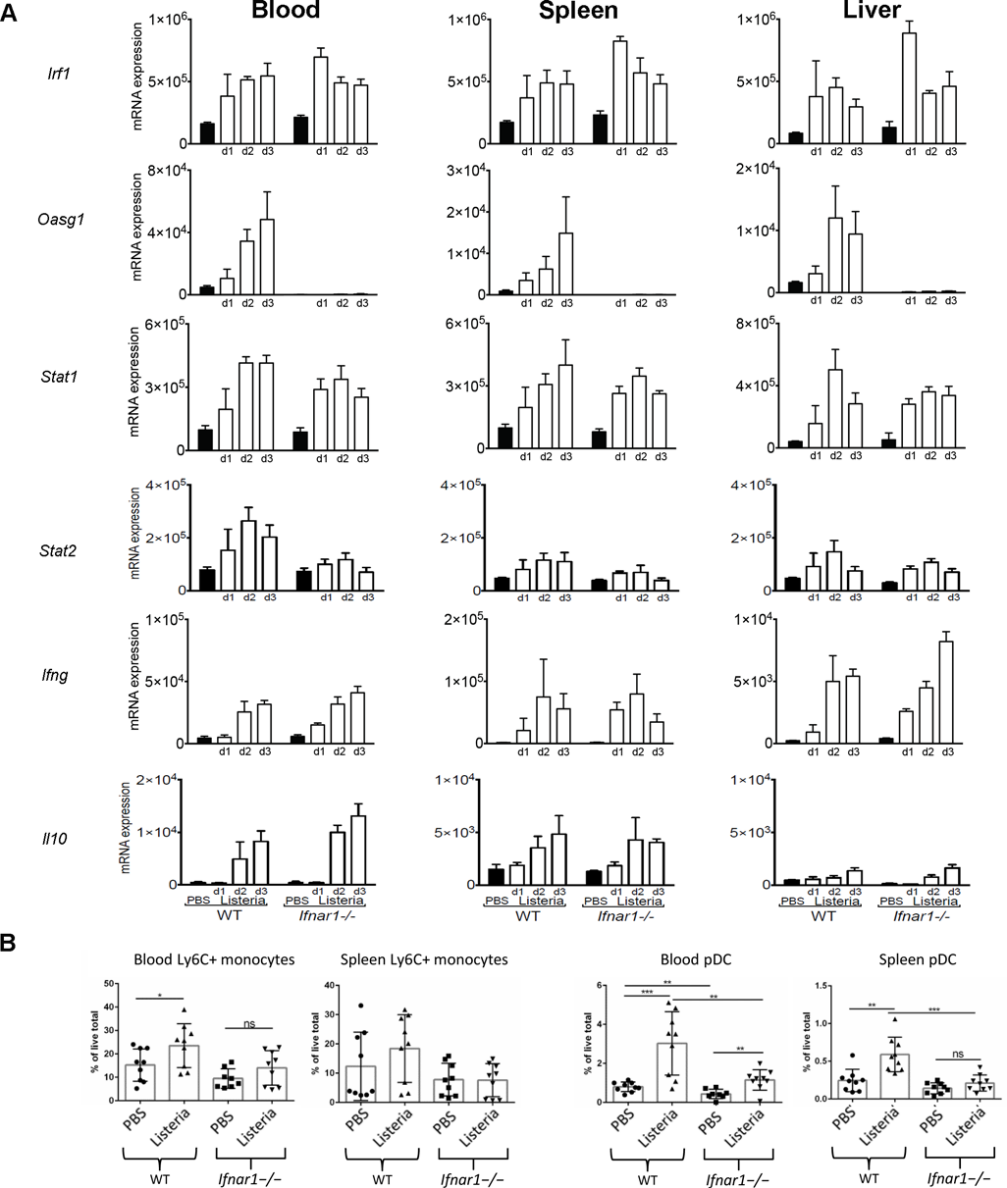
Validation of several IFN regulated genes by qRT-PCR. **(A)** qRT-PCR validation of several IFN regulated genes identified in Figs 4 and 5. Blood (left panel), spleen (middle panel) and liver (right panel) RNA was extracted at the indicated times post *L*. *monocytogenes* infection. RNA was reverse transcribed to cDNA, and expression of the indicated genes was analyzed by qRT-PCR. Values were normalized relative to *Hprt1* expression levels (mean with SD). Data are from one experiment with four mice per group. **(B)** Ly6C^+^ monocytes and pDC in blood and spleen of uninfected and infected WT and *Ifnar1*^*-/-*^ mice as a percentage of total live cells. Pooled results from 3 independent experiments, mean with SEM, n = 3 per group/experiment.

Analysis of the specific genes within the IFN signaling pathway that were differentially perturbed in the blood of WT versus *Ifnar1*^*-/-*^ infected mice showed that some genes such as *Oas1* and *Ifit3* were completely dependent on type I IFN at all time points, regardless of the bacterial load (Fig 3A) since their expression was higher in the infected WT than the infected *Ifnar1*^*-/-*^ mice at all time-points post infection (Fig 4D). In contrast, expression of other genes in the IFN signaling pathway *(G1p2*, *Ifi35*, *Ifit1 and Stat2)* were shown to be higher in the infected WT as compared to the infected *Ifnar1*^*-/-*^ mice after days 2 and 3 of infection (Fig 4D), when the bacterial load was lower in the infected *Ifnar1*^*-/-*^ mice (Fig 3A). Such changes in gene expression were validated by RT-PCR for the genes *Oasg1* and *Stat2* (Fig 6A). Although most of the perturbed transcripts within the IFN signaling (Fig 4D) and antigen presentation (Fig 4E) pathways were upregulated upon infection in both WT and *Ifnar1*^*-/-*^ mice, some transcripts such as *Ifng*, *Stat1*, *Tap1*, *Nlrc5* (Fig 4D and 4E) as well as other transcripts such as *Il10* (S1 File) were expressed at a much reduced level in the infected WT compared to the infected *Ifnar1*^*-/-*^ mice, suggesting that type I IFN signaling actually impairs the induction of these genes during infection. Such changes in gene expression were validated by RT-PCR for the genes *Ifng*, *Stat1 and Il10* (Fig 6A). The same trend of gene regulation was also observed for other transcripts from most of the top canonical pathways (S1 File).

An increase in the proportion of Ly6C^+^ monocytes was observed in the blood of WT mice upon infection but not in the blood of *Ifnar1*^*-/-*^ infected mice at day 3 post infection (Fig 6B, S4 Fig), in keeping with the observations that antigen presentation pathway at the transcriptional level was elevated in the blood of infected WT as compared to infected *Ifnar1*^*-/-*^ mice (Fig 4C). A trend towards increased Ly6C^+^ monocytes was also observed in the WT spleen upon infection but this did not reach statistical significance. Additionally proportionally there were more plasmacytoid dendritic cells (pDC) in the blood of the WT mice as compared to the *Ifnar1*^*-/-*^ both in uninfected controls and also following *L*. *monocytogenes* infection (Fig 6B) in keeping with the observations that antigen presentation pathway at the transcriptional level was elevated in the blood of infected WT as compared to infected *Ifnar1*^*-/-*^ mice (Fig 4C). In the spleen an increase in the proportion of pDC was observed in the WT and this was abrogated in the *Ifnar1*^*-/-*^ upon infection (Fig 6B).

### Canonical pathways associated with blood transcripts are differentially expressed in *Ifnar1*^−/−^ versus WT mice infected with *L*. *monocytogenes*

Since a number of transcripts appeared to be highly upregulated at day 1 post infection in the blood of infected *Ifnar1*^*−/−*^ as compared to infected WT mice where a change in expression of these transcripts over uninfected controls was not observed (Fig 3B) we performed a similar two-step filtering process as in described above for Fig 4. From the two-way ANOVA lists with the transcripts generated in Fig 3B, S2 Table, blood transcripts which this time were inducible in infected *Ifnar1*^*−/−*^ mice by at least 1.5FC upon infection were identified (Fig 5B) for each time point post infection. These lists of transcripts were then analyzed using IPA and the top 15 *p-value* ranked pathways were first identified in infected versus uninfected *Ifnar1*^*−/−*^ mice at each time point, yielding a total top 26 canonical pathways unique or shared within each of the three time points (Fig 5B). The same fold change followed by IPA analysis was also applied to blood transcripts from the WT mice upon infection this time as compared to uninfected *Ifnar1*^*−/−*^ mice and the 26 canonical pathways defined from Fig 5B were applied to the data and ranked as shown (Fig 5A). These transcripts were then further filtered to retain those which were at least 1.5FC different in blood from infected *Ifnar1*^*−/−*^ as compared to infected WT mice, all relative to blood from control uninfected *Ifnar1*^*−/−*^ mice and these transcripts were then subjected to IPA analysis (Fig 5C). This identified the final net difference in the maximal gene expression change upon infection in the *Ifnar1*^*-/-*^ as compared to the WT mice having taken into account the *Ifnar1*^*-/-*^ baseline difference.

These data largely corroborated the data shown in Fig 4A–4C, with a dominance of the IFN signaling and antigen presentation pathways, where the transcripts in this case were more highly up-regulated in the blood of the *Ifnar1*^*-/-*^ as compared to the WT mice at day 1 post infection, but down-regulated at days 2 or 3 post infection (Fig 5C). This was reciprocal to data in Fig 4 supporting differential temporal role of type I IFN in these pathways. However, additional pathways was revealed to be perturbed, for example up-regulation of the TREM 1 signaling pathway in the infected *Ifnar1*^*-/-*^ as compared to the infected WT mice, was observed (Fig 5C). Furthermore, the role of type I IFN signaling was confirmed by this approach for genes including *Oas1*, *Ifit3 and Ifng*, but additionally *Irf9* was shown to be higher (Fig 5D) in the infected *Ifnar1*^*-/-*^ as compared to the infected WT mice at day 1 post infection when the bacterial loads were similar (Fig 3A), but lower at day 3 in the infected *Ifnar1*^*-/-*^ when the bacterial loads were reduced (Fig 3A). These results were validated for *Oas and Ifng* by RT-PCR (Fig 6A). Within the antigen presentation pathway in addition to genes revealed to be down-regulated by type I IFN in Fig 4E (*Ifng*, *Tap1* and *Nlrc5)*, *Lmp2* was also now revealed to be regulated by type I IFN, since its expression was shown to be higher in infected *Ifnar1*^*-/-*^ as compared to the infected WT mice (Fig 5E). The same trend of gene regulation was also observed for other transcripts from most of the top canonical pathways (S2 File).

### Absence of type I IFN signaling results in differences in baseline expression of IFN inducible genes

**Fig 7:**
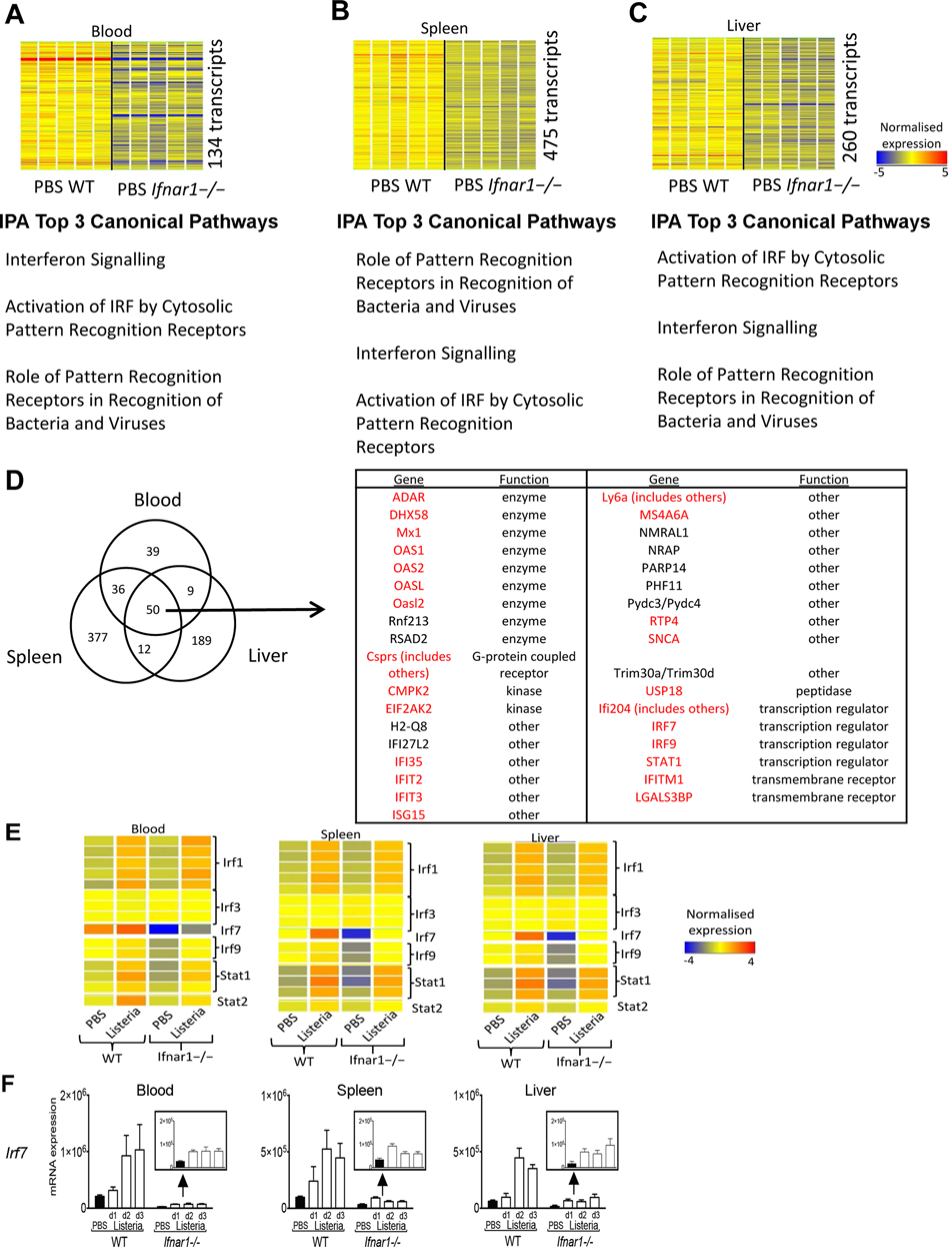
*Ifnar1*^-/-^ uninfected mice show a lowered expression of several IFN regulated genes as compared with WT uninfected mice. **(A–C)** The strain-associated subsets of transcripts identified from the 2-way ANOVA as in Fig 3B for an independent experiment were filtered to identify baseline differences (fold-ratio of 1.5 between day 3 uninfected *Ifnar1*^**-/-**^ to day 3 uninfected WT mice) for blood, spleen and liver. Heatmaps and a list of top three IPA® canonical pathways are shown. **(D)** Venn diagram of above detailed transcripts identifies 50 transcripts that are commonly shared between blood, spleen and liver. These transcripts map to 35 genes in IPA® and include a number of Interferome-based type I IFN responsive genes that are marked in red. **(E)** Heatmap of mean-normalised expression values for selected (*Irf1*, *Irf3*, *Irf7*, *Irf9*, *Stat1* and *Stat2*) IFN transcriptional regulator transcripts. **(F)** qRT-PCR validation of *Irf7* gene normalized relative to *Hprt1* gene in blood (left panel), spleen (middle panel) and liver (right panel) at indicated times post infection (mean with SD). Data from one experiment with four mice per group.

Having observed that there was differential transcript expression in the blood of uninfected *Ifnar1*^*−/−*^ compared to the uninfected WT mice (Fig 3C and data not shown from independent experiment using the same methods as Fig 3C, which validated the findings) we set out to identify transcripts whose expression was at least 1.5FC lower in the uninfected *Ifnar1*^*−/−*^ compared to the uninfected WT group using the same analysis method as in Fig 3C (Fig 7A–7C and data not shown from an independent experiment which validated our findings). These 134, 475 and 260 transcripts in the blood, spleen and liver respectively were enriched for IFN related pathways when analysed by IPA canonical pathway analysis (Fig 7A–7C). Identifying the common transcripts across the three tissues revealed 50 transcripts, which comprised 35 unique genes (using IPA annotation) of which 25 were identified as being type I IRGs from the Interferome v2 database (Fig 7D). These 25 genes included members of the 2',5'-oligoadenylate synthetase (OAS) family, IFN regulatory factors *Irf7* and *Irf9* as well as *Stat1*, which were at least 1.5 FC lower even in the absence of infection.

Focusing on the expression of selected key IFN signaling genes (*Irf1*, *Irf3*, *Irf7*, *Irf9*, *Stat1*, *Stat2*; Fig 7E), we found firstly that *Irf1* has similar baseline expression in WT and *Ifnar1*^*-/-*^ mice, is induced by a similar degree upon infection and therefore its final expression is comparable between the mouse strains following infection. *Irf3*, which is constitutively expressed [31] was not affected by either IFNαβR deficiency or infection. *Irf7* expression was at least 1.5 FC lower already at baseline, and although induced by an equivalent degree upon infection their final expression remained significantly lower than that in WT mice following infection. These results were validated by RT-PCR (Figs 6A and 7F). Expression of *Irf9* was seen to be much lower in *Ifnar1*^*-/-*^ mice as compared to WT at the basal level. Despite having observed that the induction of *Irf9* was greater in the *Ifnar1*^*-/-*^ compared to the WT following infection in all three tissues, the final expression values of *Irf9* remained lower in the *Ifnar1*^*-/-*^ mice following infection than that observed in WT mice even before infection (Fig 7E).

### Absence of type I IFN signaling leads to enhanced or reduced type I and type II IFN responsive gene expression at day 1 or day 3 post *L*. *monocytogenes* infection respectively

**Fig 8:**
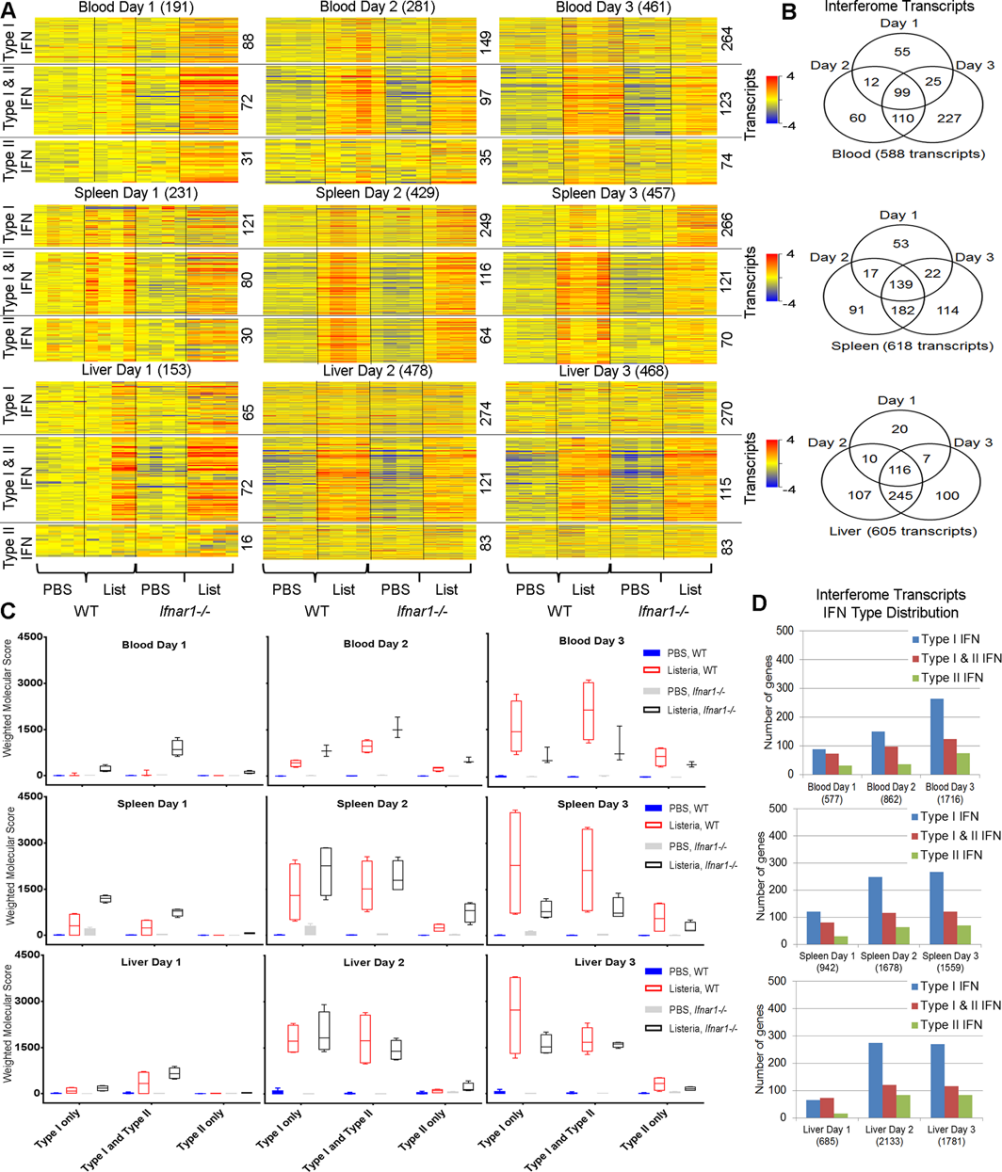
IFN response genes show differential early and delayed expression patterns following *L*. *monocytogenes* infection in WT and *Ifnar1*^-/-^ mice. **(A)** Heatmaps of IFN-response genes (type I, type II, and type I and II) associated with blood, spleen and liver transcripts reported in Fig 3B and the Interferome database are shown. Total numbers of IFN-response genes identified across each of the three tissues and their time-course distribution are shown at far right. **(B)** Venn diagrams showing temporal differences and similarities in the expression of IFN-response genes in blood, spleen and liver. **(C)** Weighted molecular scores of the identified IFN-response genes calculated relative to uninfected WT mice. **(D)** The distribution of IFN-response genes within blood, spleen and liver of infected and uninfected mice at individual times post infection.

Using the Interferome v2 database [30] we identified from our significant transcript lists (Fig 3B and S1 Table) genes that were classified as either type I IFN responsive, type II IFN responsive or both type I and type II IFN responsive (Fig 8A and S2 Table). Statistical filtering (2-way ANOVA with Benjamini Hochberg multiple testing correction, *P* <0.05) revealed 588, 618 and 605 significantly differentially expressed IFN responsive transcripts in blood, spleen and liver during the course of infection (i.e. days 1, 2 and 3) which were differentially expressed in infected and uninfected *Ifnar1*^*-/-*^ or WT mice (Fig 8A and 8B). It should be noted that there was perturbation of a larger number of transcripts at days 2 and 3 than day1 post-infection in blood and tissues of both *Ifnar1*^*-/-*^ and WT mice. Although unique transcripts were perturbed at each of the different time points post infection, some common transcripts between time points were also observed (Fig 8B). For all three tissues the majority of IFN responsive transcripts identified were type I or both type I and II IFN responsive genes at each time point post infection, with a marked increased of type I IFN responsive transcripts that are differently regulated upon infection over time (Fig 8A and 8D).

Strikingly, an upregulation of a significant number of those transcripts was observed at day 1 post infection in blood of the *Ifnar1*^*-/-*^ as compared to WT mice (Fig 8A), whereas the opposite was observed at day 3 post infection where decreased transcriptional expression was observed (Fig 8A). In order to quantify expression we calculated a weighted molecular score, derived by previously published methods [32], of these transcripts. The expression of transcripts classified as type I, both type I and type II as well as type II IFN responsive was elevated at day 1 (where bacterial loads are similar, Fig 3A), and diminished at day 3 (when the bacterial loads are reduced in the infected *Ifnar1*^*-/-*^ mice, Fig 3A) in the *Ifnar1*^*-/-*^ mice compared to the WT mice in the blood, spleen and liver following *L*. *monocytogenes* infection (Fig 8C), demonstrating how IFN responsive genes are differentially affected at different time points after infection.

## Discussion

Transcriptomic analyses of expressional changes have great potential in the study of global responses to a pathogen and for the understanding of what constitutes an effective versus a suboptimal or detrimental immune response [26–28, 33, 34]. When studying the immune response in humans, researchers may have access only to the blood, and not the tissues where the ongoing infection or autoimmune processes are occurring. We demonstrate herein that gene expression changes observed in the tissue upon infection with the intracellular bacteria *L*. *monocytogenes* also occur in blood, demonstrating the utility of transcriptional analysis of blood. In addition, we show that different changes in the gene expression profile in blood and tissues in *Ifnar1*^*-/-*^ as compared to WT mice are observed at varying times post-infection. In addition we show that genes are not only differentially expressed upon infection but also in the uninfected state in *Ifnar1*^*-/-*^ as compared to WT mice, which likely contributes to the net global expression changes, which we observe after infection. This may be explained by either changes in gene transcription in discrete cell types or changes in cell composition, such T cells, myeloid cells and plasmacytoid DC.

We show firstly that the immune-inflammatory transcriptional response during *L*. *monocytogenes* infection in a murine model system, as seen in whole blood, relates well to that occurring at major sites of infection such as the spleen. Upon infection with *L*. *monocytogenes* increases and decreases in gene expression were observed which were enriched for TREM1, IL-10 and Granulocyte Adhesion and diapedesis some of which are in keeping with previously reported mechanisms of protection or pathogenesis [3, 8, 16, 23, 24, 35, 36]. In addition, differential expression of a large number of type II IFN-inducible genes was observed, which are known to be important for protection in the immune response against this intracellular pathogen, in part through activation of phagocytosis and bacterial killing in macrophages [3, 8, 35]. Additional changes in expression of genes known to be downstream of type I IFN signaling, which is known to prevent bacterial control [14–16] were also shown to be enriched in both blood and spleen of infected mice. This is in keeping with previous reports on the cross-regulation between these two IFN signaling pathways [20, 37].

Down-regulation of lymphocyte signaling pathways were observed in the spleen and the blood of infected mice, which could be accounted for by a reduced proportion of T cells that we observed by flow cytometric analysis of the blood and spleen of infected mice. Previous findings have shown that *L*. *monocytogenes* infection induces the apoptosis of lymphocytes [23, 38, 39], which may reflect this observed reduction in the relative percentage of T-cells in the blood and spleen and downregulation of T-cell signaling pathways observed at the transcriptional level. Although we could not see a difference in expression levels of general apoptotic genes this could be since at this time point post infection the cells expressing the apoptotic genes had already apoptosed as our flow cytometry data supports. This demonstrates the usefulness of blood transcriptional profiling to capture the global response to pathogens to reveal potential mechanisms of protection or pathogenesis in both mouse and man.

We sought to investigate further how abrogation of type I IFN signaling would impact blood and tissue transcriptional signatures occurring during *L*. *monocytogenes* infection using *Ifnar1*^*-/-*^ mice, to shed additional light on how type I IFN may negatively regulate bacterial clearance. The majority of transcripts which were identified to be significantly differentially expressed in WT and *Ifnar1*^*-/-*^ strains of mice were changed as a result of the infection at days 2 and 3 post-infection. A smaller proportion of transcripts were significantly differentially expressed as a result of the combination of infection and WT and *Ifnar1*^*-/-*^ mouse strains at days 2 and 3 post-infection, where there was a significant difference in bacterial loads. By comparing the fold change response of transcripts to infection in WT or *Ifnar1*^*-/-*^ mouse strains, it was seen that the response was broadly comparable in the WT and *Ifnar1*^*-/-*^ despite the absence of type I IFN signaling. This also included the majority of genes found within the IFN IPA signaling pathway. Of note, the perturbation of blood transcripts at day 1 was most pronounced in WT and *Ifnar1*^*-/-*^ mice as a result of infection (approximately 42%), at a time-point when there was no difference in bacterial loads. Of the 42% of transcripts that were perturbed at day 1 post infection in the blood of *Ifnar1*^*-/-*^ as compared to the WT mice, detailed IPA analysis showed increased expression of genes associated with antigen presentation, including *Tap1*, *Nlrc5*, *Lmp2*, and increased expression of *Ifng*, would suggest enhanced antigen presentation in the absence of type I IFN signaling. In addition increased levels of *Ip10*, *Irf1*, *Stat1*, *Icsbp*, at day 1 post infection in the blood of *Ifnar1*^-/-^ as compared to the WT mice is in keeping with type I IFN inhibition of Th1 responses and could explain the subsequent reduction in bacterial load in the *Ifnar1*^-/-^ infected mice. Our findings that increased *Ifng* expression was accompanied by increased levels of *Il10* could suggest that Th1 cells which have been shown to produce both cytokines [40] are reduced in the WT possibly by type I IFN inhibition of IL-12 production by myeloid cells as we have previously demonstrated during *Mycobacterium tuberculosis* infection [41].

Changes in gene expression were also seen in the blood of the *Ifnar1*^*-/-*^ as compared to WT mice in the absence of infection (approximately 25%), suggesting that type I IFN signaling is already evident in uninfected mice. The small number of transcripts shown to be perturbed in the blood and tissue of *Ifnar1*^*-/-*^ as compared to WT mice in the absence of infection were shown a cohort of genes dependent on IFNαβR signaling. A proportion of these type I IRGs match known type I IFN responsive genes as identified in the Interferome v2 database. Amongst these genes dependent on IFNαβR signaling in the uninfected mice were IRGs including OAS family genes, IRF genes, and *Stat1*. We found that many of these same genes had similar FC induction following infection, however given the lower expression in the uninfected *Ifnar1*^*-/-*^ compared to the WT their final expression remained lower in the infected *Ifnar1-/-* compared to the WT. This finding highlights the need to consider both the relative expression levels as well as the degree of gene induction (fold change) when interpreting expression data. Basal levels of *stat1* gene expression are reduced in the blood and tissue of uninfected *Ifnar1*^*-/-*^ mice as compared to WT controls. This effect on *Stat1* in uninfected *Ifnar1*^*-/-*^ mice has previously been described at both the mRNA and protein level in a variety of tissues [42]. IFN-γ and type I IFNs require STAT1 for signaling [43]. IFN-γ utilises the STAT1 homodimer (GAF) whereas type I IFNs utilise the heterotrimer of STAT1-STAT2 and IRF9 (ISGF3) to initiate transcription at their respective promoter sites [43]. This lower *Stat1* expression may explain in our system the observation of diminished type II IFN inducible gene expression in the *Ifnar1*^*-/-*^ compared to the WT following infection.

Previous evidence has suggested that low-level type I IFN signaling occurs in the steady state [44], and this type I IFN priming may be important for optimal responses to cytokines and cytokine production from immune cells [42, 45]. Similarly, the commensal gut microbiota has been shown to signal in part via the type I IFN receptor and this signaling is important for the priming of the immune system and its subsequent response to pathogens [46–48]. Our findings suggest that this basal priming of type I IFN signaling may contribute to the overall response to a pathogen and its resolution. Expression of *Irf7*, which is known to be one of the most inducible *Irf* genes [31] was induced in the *Ifnar1*^*-/-*^ upon infection to an equivalent degree as compared to the WT. However, its basal level of expression in *Ifnar1*^*-/-*^ mice was so low to start with that its final expression in blood and tissue upon infection remained lower than that seen in the WT mice before infection. Thus, although clearly inducible in the absence of type I IFN signaling (it can be induced by other factors [49]), it is clear that despite this the magnitude of the response is limited by basal priming by Type I IFN. In contrast *Irf3* expression was not affected by either infection or the absence of Type I IFN signaling. Expression of *Irf1* was increased in response to infection but in contrast to *Irf7*, *Irf9* and *Stat1*, there was no difference in baseline or final expression in response to infection in WT and *Ifnar1*^*-/-*^ mice.

The expression levels of both type I and type II IFN inducible genes following infection as shown by the Molecular Distance to Health (MDTH) was reduced in the *Ifnar1*^*-/-*^ compared to the WT mice at day 3 post infection where the bacterial load was much diminished in the infected *Ifnar1*^*-/-*^ mice. Thus this decrease in type I and type II IFN inducible transcripts may result from either a dependence on type I IFN signaling or a lower bacterial load. However, at day 1 post infection where the bacterial load was not affected in the infected *Ifnar1*^*-/-*^ the converse was true with an increase in both type I and type II IFN-inducible genes suggesting a direct role of type I IFN signaling in suppression of this response. Previous studies have shown that type I IFN production during *L*. *monocytogenes* infection suppresses macrophage activation by IFN-γ, by decreasing *Ifngr1* mRNA and surface expression of the IFN-γ receptor [25]. At the whole tissue level we do not see significant *Ifngr1* expression above background levels in either WT or *Ifnar1*^*-/-*^ mice (data not shown), which may highlight the complexities of studying multiple cell types within tissues in transcriptomic approaches. We observed increased expression of *Stat1* in the blood of infected *Ifnar1*^*-/-*^ mice versus WT controls at day 1 post infection when bacterial loads were similar, suggesting a direct of role of type I IFN signaling. However at day 3 post infection diminished expression of *Stat1* was observed across all three tissues, following infection in the *Ifnar1*^*-/-*^ compared to the WT mice, which may be explained by a dependence on type I IFN signaling or may result from the lower bacterial load in the *Ifnar1*^*-/-*^.

The increase in the proportion of Ly6C^+^ monocytes following *L*. *monocytogenes* infection that we observe in the blood and spleen of WT mice is abrogated in the *Ifnar1*^*-/-*^ mice probably as a result of the loss of IFN-inducible genes known for increasing numbers of Ly6C^+^ monocytes. We also observed a significant decrease in pDC in the blood of uninfected *Ifnar1*^*-/-*^ mice compared to uninfected WT mice. Following infection there was a greater relative percentage of pDC in both the blood and the spleen of the WT mice compared to the *Ifnar1*^*-/-*^ mice. This observation may be due to the requirement of type I IFNs for pDC activation, migration and survival as it has been previously shown [50–52].

This study highlights the utility of using whole blood transcriptional signatures to profile the host transcriptional response in a model of infection. We show that the blood, spleen and liver signatures share similarity in their transcriptional response following infection and that these derived signatures are enriched for both type I and type II interferon signaling but differ according to the stage of infection. The role of type I and type II interferons in *L*. *monocytogenes* infection is complex with type II interferon signaling affording protection while type I interferon signaling promotes bacterial survival. This study also shows that the absence of type I IFN signaling alters the expression of multiple genes at the whole tissue level in uninfected mice. This lower expression may impact upon genes involved in more than one signaling pathway, the result of which in an *in vivo* model of *L*. *monocytogenes* infection can result in a complex perturbation. These findings highlight the need to consider both the relative expression levels as well as the degree of gene induction when interpreting expression data and the temporal changes in gene expression, particularly in genetically manipulated cell lines or animals where the basal expression in a number of genes may be altered.

## Materials and Methods

### Ethics statement

All research was performed in strict accordance with the Animals (Scientific Procedures) Act, 1986 and compliance was monitored closely by the Francis Crick Institute’s Ethical Review Committee and the UK Home Office.

### Mice

Female C57BL/6 mice and *Ifnar1*^*-/-*^ mice (backcrossed onto the C57BL/6 background) were bred and housed in the specific pathogen-free facilities at The Francis Crick Institute, Mill Hill Laboratory, London, UK. Mice were 8 weeks of age at the start of experiments.

### Bacteria and infection of mice

Bacteria were grown in brain heart infusion (BHI) broth (BD BBL) to mid-log phase as determined by OD_560_ measurement and aliquots were stored in 20% glycerol/phosphate-buffered saline (PBS) at –80°C. For infection, mice received 5 × 10^3^ colony-forming units (CFU) *L*. *monocytogenes* intravenously in 0.2 ml Dulbecco’s PBS (Gibco), or PBS alone. The actual number of bacteria injected was verified by plating aliquots of the relevant inoculum onto BHI agar (BD BBL). Bacterial growth in the spleen and liver was determined at day 1, 2 or 3 post infection by plating serial dilutions of spleen and liver homogenates from animals that were not used for transcriptional studies. Colonies were counted after 24 h of incubation at 37°C. Graphs were generated in GraphPad Prism 6.

### Collection and purification of RNA from tissues

Infected and uninfected control mice were sacrificed at day 1, 2, or 3 post *L*. *monocytogenes* infection. Approximately 0.5 ml whole blood were collected by intracardiac puncture and immediately mixed vigorously with 1 ml Tempus solution (Applied Biosystems) before being stored at –80°C until RNA extraction. Spleens and livers were removed aseptically and were homogenized immediately in TRIReagent® RNA buffer (Ambion/Life Technologies) by pulsing with a Polytron PT1600E homogenizing unit (Kinematic) before being frozen at –80°C. Blood total RNA was isolated using approximately 0.25 ml collected blood (thus 0.75 ml blood/Tempus solution) and the PerfectPure RNA Blood kit (5PRIME) according to the manufacturer’s instructions. Globin RNA was removed from 2 μg of isolated total RNA using the GLOBINclear™ mouse/rat whole blood reduction kit (Ambion/Life Technologies) according to the manufacturer’s instructions. For spleen/liver total RNA extraction, samples were thawed and the RiboPure kit (Ambion/Life Technologies) was used according to manufacturer’s instructions. RNA quantities were measured using either a NanoDrop 1000 or 8000 spectrophotometer (NanoDrop Products/ThermoScientific). RNA integrity was assessed using either an Agilent 2100 Bioanalyzer (Agilent Technologies), or LabChip GX (Caliper Life Sciences/Perkin Elmer). Samples with an RNA integrity number >7 were retained for further processing.

### Gene expression profiling

Biotinylated, amplified antisense complementary RNA (cRNA) targets were prepared from 300 ng of either globin-reduced blood RNA, purified spleen total RNA, or purified liver total RNA, using the Illumina® CustomPrep RNA amplification kit (Applied Biosystems/Ambion). For each sample, 1.5 μg of labelled cRNA was hybridized overnight to Illumina® MouseWG-6 v2.0 Expression BeadChip whole genome arrays (Illumina Inc., San Diego, USA; www.illumina.com), which contained more than 45,000 probes. The arrays were then washed, blocked, stained, and scanned on an Illumina iScan, following the manufacturer’s instructions. Genome Studio software (Illumina) was used to generate signal intensity values, quality control values, and to subtract background values.

### Data analysis

#### Agilent Technologies Inc. GeneSpring GX v12.1 analysis

Background subtracted microarray data were processed and analysed using GeneSpring GX software version 12.1 (Agilent Technologies Inc.; www.agilent.com). Each probe was attributed a flag to denote its signal intensity detection *p*-value. These flags were used to filter out probes that did not result in a ‘present’ call in at least 10% of the samples of a particular dataset (‘present’ lower cut off set to 0.99). Data were corrected for low signal values by thresholding values less than 10 to 10, log_2_-transformed, and normalized to the 75^th^ percentile. Values were then baseline transformed to the median of all samples. Entities were filtered by expression level to keep transcripts with a detection *p*-value cut-off > 0.01 and with a two-fold difference from the median in at least 10% of all samples. Differentially expressed transcripts were identified by either unpaired *t*-test (*p* < 0.01) or 2-way ANOVA (*p* < 0.05), each with Benjamini–Hochberg false discovery rate (FDR) correction on transcripts passing the above described QC filtering. Heatmaps for differentially expressed genes were clustered on entities applying hierarchical Pearson’s uncentred average linkage rule. Venn diagrams were generated in GeneSpring GX. Bar charts were generated in Microsoft® Excel. Microarray data were deposited in the NCBI Gene Expression Omnibus (GEO) with series accession number (GSE77102). All data collected and analyzed in these experiments adhere to the Minimal Information About a Microarray Experiment (MIAME) guidelines.

#### QIAGEN’s Ingenuity® Pathway Analysis

Data were analysed through the use of QIAGEN’s Ingenuity® Pathway Analysis (IPA®, QIAGEN Redwood City, CA, USA; www.qiagen.com/ingenuity). Canonical pathway analysis identified the most significantly represented pathways in the microarray datasets (Fisher’s Exact test *p* < 0.05 with Benjamini–Hochberg FDR correction). Data analyses and heatmaps in Figs 4 and 5 and S1 and S2 Files were generated in Microsoft® Excel from IPA output files.

#### Interferome database analysis and weighted molecular score

IFN response genes (IRGs) (type I, type II, and type I and II) listed in the Interferome database (release v2.01; www.interferome.org accessed September 2015) were quantitated. Weighted molecular scores of the IRGs were calculated relative to the uninfected WT (PBS treated) for three sample groups (WT infected, PBS treated *Ifnar1*^*-/-*^, and infected *Ifnar1*^*-/-*^ mice). In detail, raw expression values for differentially expressed transcripts labelled as IRGs in the Interferome database were input to Microsoft® Excel and the number of total transcripts was adjusted to reflect the respective list size. Data analysis, heatmaps, Venn diagrams and bar charts were generated using either GeneSpring GX, Microsoft® Excel or GraphPad Prism 6 software.

### Quantitative PCR analysis

qRT-PCR was performed on selected transcripts. RNA was reverse transcribed with a high-capacity reverse transcription kit (Applied Biosystems) to cDNA. The expression of indicated genes was quantified by real-time PCR (ABI Prism 7900 from Applied Biosystems) and normalized against *Hprt* mRNA expression levels (mean with SD, *n* = 3–5 mice/group). Bar charts were generated in GraphPad Prism 6.

### Flow cytometry

Three independent experiments with 3 mice per group per experiment were undertaken. Blood was harvested by cardiac puncture and red cells removed prior to FACS staining by incubation with ACK red cell lysis buffer. Spleen single cells suspensions were made by gently mashing organs through 70 μm sieves using the plunger of a syringe. Spleen suspensions were also subjected to red cell lysis prior to FACS staining. Cell suspensions were washed with PBS and incubated with 2 μg anti-CD16/CD32 (clone 2.4G2, NIMR). To identify live cells, cells were stained with a live/dead stain kit (Invitrogen). Extracellular markers were identified by staining with monoclonal antibodies against CD3, CD4, CD8, CD49b, F4/80, CD19, CD11c, CD11b, Ly6C, Ly6G, Siglec H, MHCII, Thy1. All antibodies were purchased from eBioscience or Biolegend. Data were analysed using Flow Jo version 9.4.10 (Treestar). Data from all 3 experiments were pooled for statistical analysis.

## Supporting Information

S1 Fig Related to Fig 1. **Spleen CFU and Principle component analysis of blood and spleen transcripts passing QC filtering for uninfected and *L*. *monocytogenes* infected C57BL/6 mice.** **(A)** C57BL/6 mice were infected intravenously with 5x10^3^ colony forming units (CFU) of *L*. *monocytogenes*. After 3 days, the bacterial load was determined in the spleen and presented as CFUs. **(B and C)** Principal component analysis of transcripts significantly detected from background (*P* < 0.01) separates infected from non-infected in **(B)** blood and **(C)** spleen. **(D)** Total number of upregulated and downregulated significantly expressed transcripts in blood and spleen following *L*. *monocytogenes* infection (from Fig 1A and 1B).
(TIF)

S2 Fig Related to Fig 1. **Blood and spleen flow cytometry analysis to identify CD3**^**+**^**Thy1.2**^**+**^ **cells.** Representative images of the gating strategy to identify subpopulations of lymphoid cells from whole blood and spleen from *L*. *monocytogenes* infected mice.
(TIF)

S3 Fig Related to Fig 3B. **Principle component analysis of blood, spleen and liver transcripts passing QC filtering for uninfected and *L*. *monocytogenes* infected WT and *Ifnar1***^***-/-***^ **mice.** Principal component analysis of transcripts significantly detected from background (*p* <0.01) separates infected from non-infected in blood, spleen and liver.
(TIF)

S4 Fig Related to Fig 6. **Flow cytometry to identify Ly6C**^**+**^ **monocytes and pDCs in blood and spleen.** Representative images of the gating strategy to identify sub populations of myeloid cells from whole blood and spleen from *L*. *monocytogenes* infected mice.
(TIF)

S1 File Related to Fig 4. **Top blood canonical pathways associated with transcripts that are differentially expressed in *L*. *monocytogenes* infected WT versus *Ifnar1***^**-/-**^ **mice relative to uninfected WT mice.** Detailed gene heatmaps for all 30 IPA top pathways from Fig 4 except interferon signaling and antigen presentation.
(PDF)

S2 File Related to Fig 5. **Top blood canonical pathways associated with transcripts that are differentially expressed in *L*. *monocytogenes* infected *Ifnar1***^**-/-**^ **versus WT mice relative to uninfected *Ifnar1***^**-/-**^ **mice.** Detailed gene heatmaps for all 26 IPA top pathways from Fig 5 except interferon signaling and antigen presentation.
(PDF)

S1 Table Related to Fig 3B. **Transcripts associated with tissue-specific responses of WT and *Ifnar1***^**-/-**^ **mice in blood, spleen and liver following *L*. *monocytogenes* infection at different time points and bacterial loads.** Columns show Illumina Probe_Id (column A), log fold change expression between infected and uninfected WT mice (column B), log fold change expression between infected and uninfected *Ifnar1*^−/−^ mice (column C), and Entrez Gene Symbol (column D).
(XLSX)

S2 Table Related to Fig 8A. **IFN response genes associated with *L*. *monocytogenes* infection in WT and *Ifnar1***^**-/-**^ **mice.** Columns show Illumina Probe_Id (column A), log fold change expression between infected and uninfected WT mice (column B), log fold change expression between infected and uninfected*Ifnar1*^-/-^ mice (column C), and Entrez Gene Symbol (column D).
(XLSX)
