# Supplementary figures and images for: Analysis of Transcriptional Signatures in Response to Listeria monocytogenes Infection Reveals Temporal Changes That Result from Type I Interferon Signaling
Source: PLoS One. 2016 Feb 26;11(2):e0150251. doi: 10.1371/journal.pone.0150251 (PMC4768944; doi:10.1371/journal.pone.0150251)

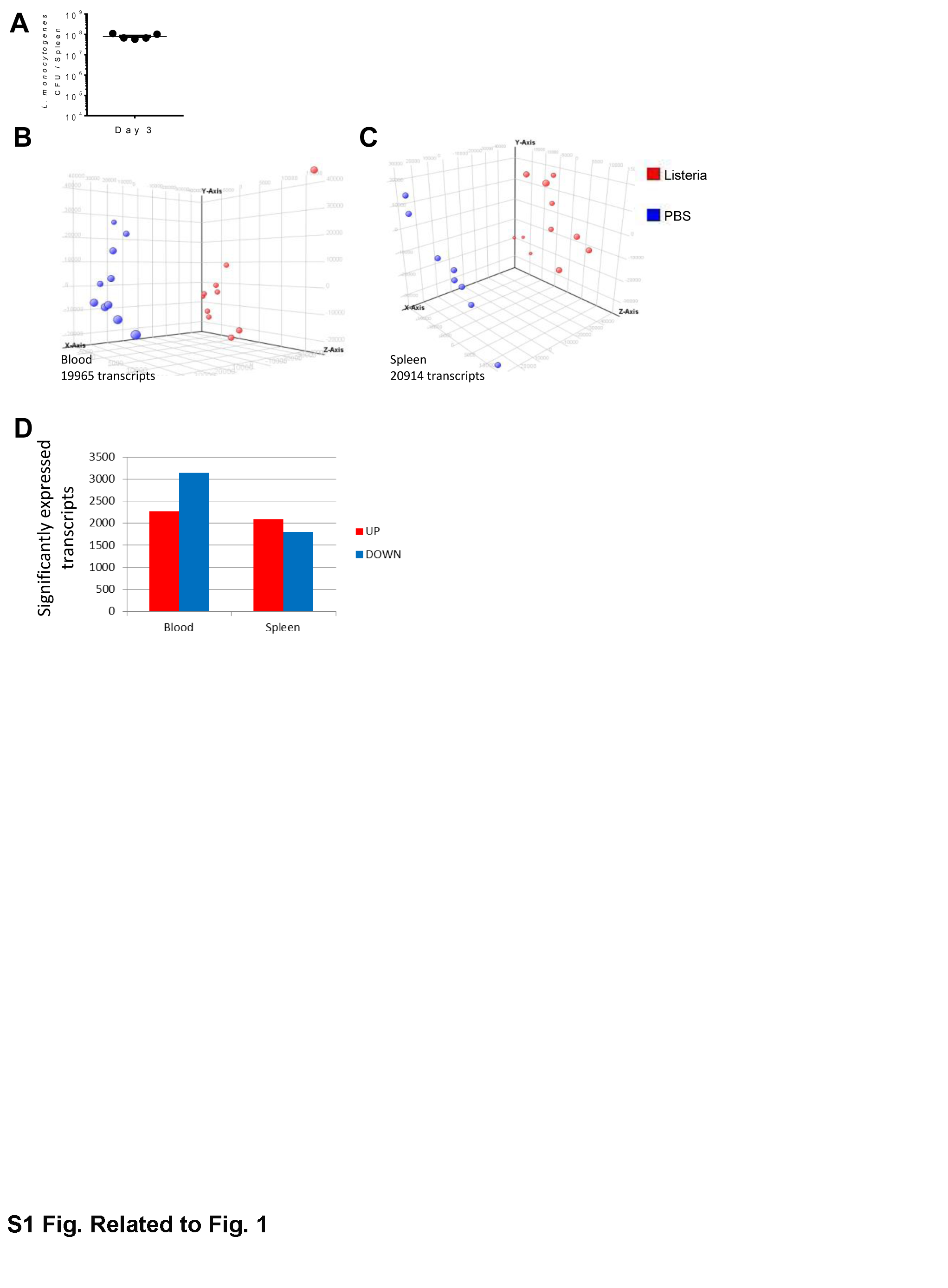

Supplement: S1 Fig — Spleen CFU and Principle component analysis of blood and spleen transcripts passing QC filtering for uninfected and L. monocytogenes infected C57BL/6 mice. (A) C57BL/6 mice were infected intravenously with 5x10^3^ colony forming units (CFU) of L. monocytogenes. After 3 days, the bacterial load was determined in the spleen and presented as CFUs. (B and C) Principal component analysis of transcripts significantly detected from background (P < 0.01) separates infected from non-infected in (B) blood and (C) spleen. (D) Total number of upregulated and downregulated significantly expressed transcripts in blood and spleen following L. monocytogenes infection (from Fig 1A and 1B). (TIF) [file pone.0150251.s001.tif]

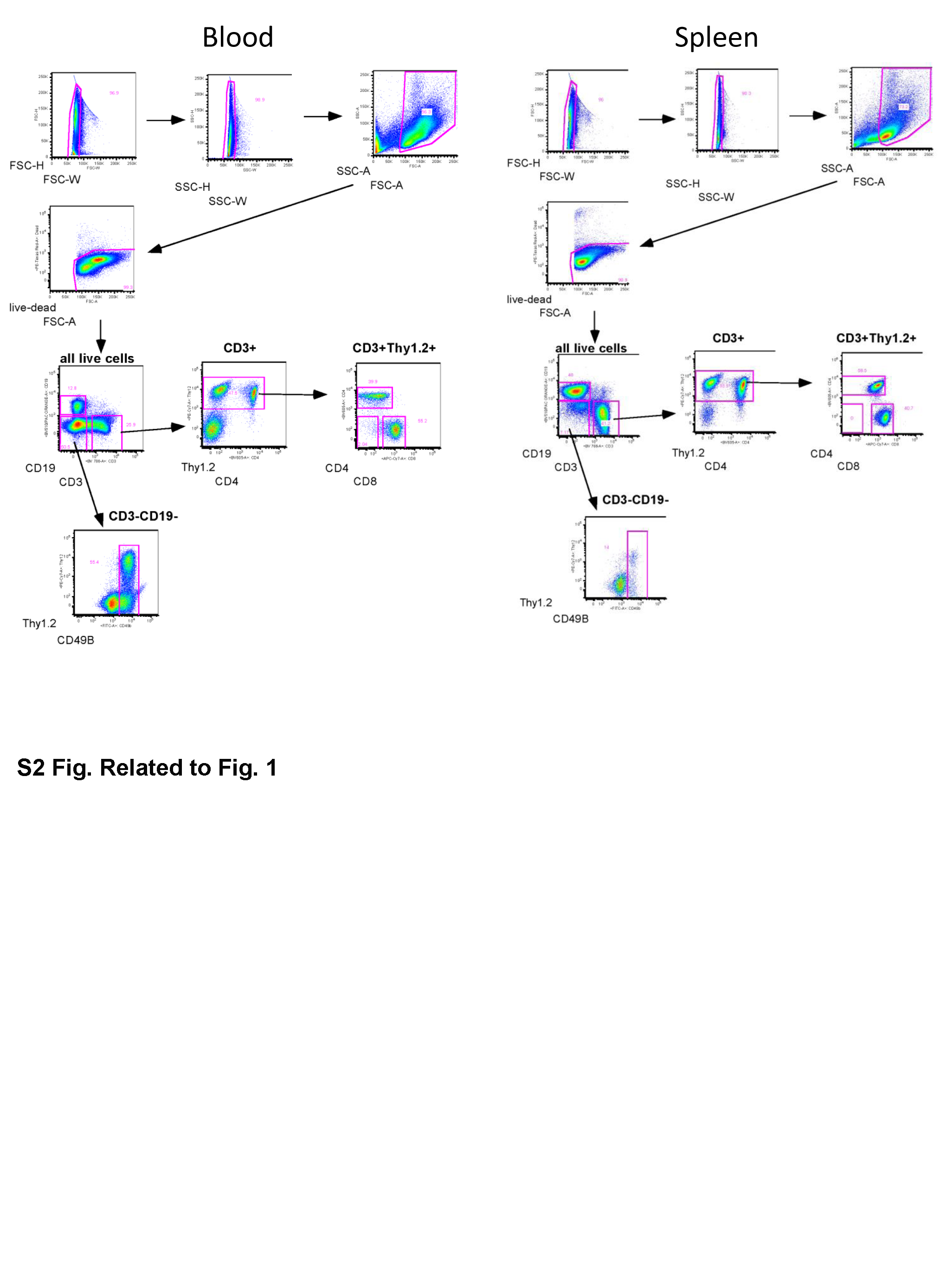

Supplement: S2 Fig — Blood and spleen flow cytometry analysis to identify CD3+Thy1.2+ cells. Representative images of the gating strategy to identify subpopulations of lymphoid cells from whole blood and spleen from L. monocytogenes infected mice. (TIF) [file pone.0150251.s002.tif]

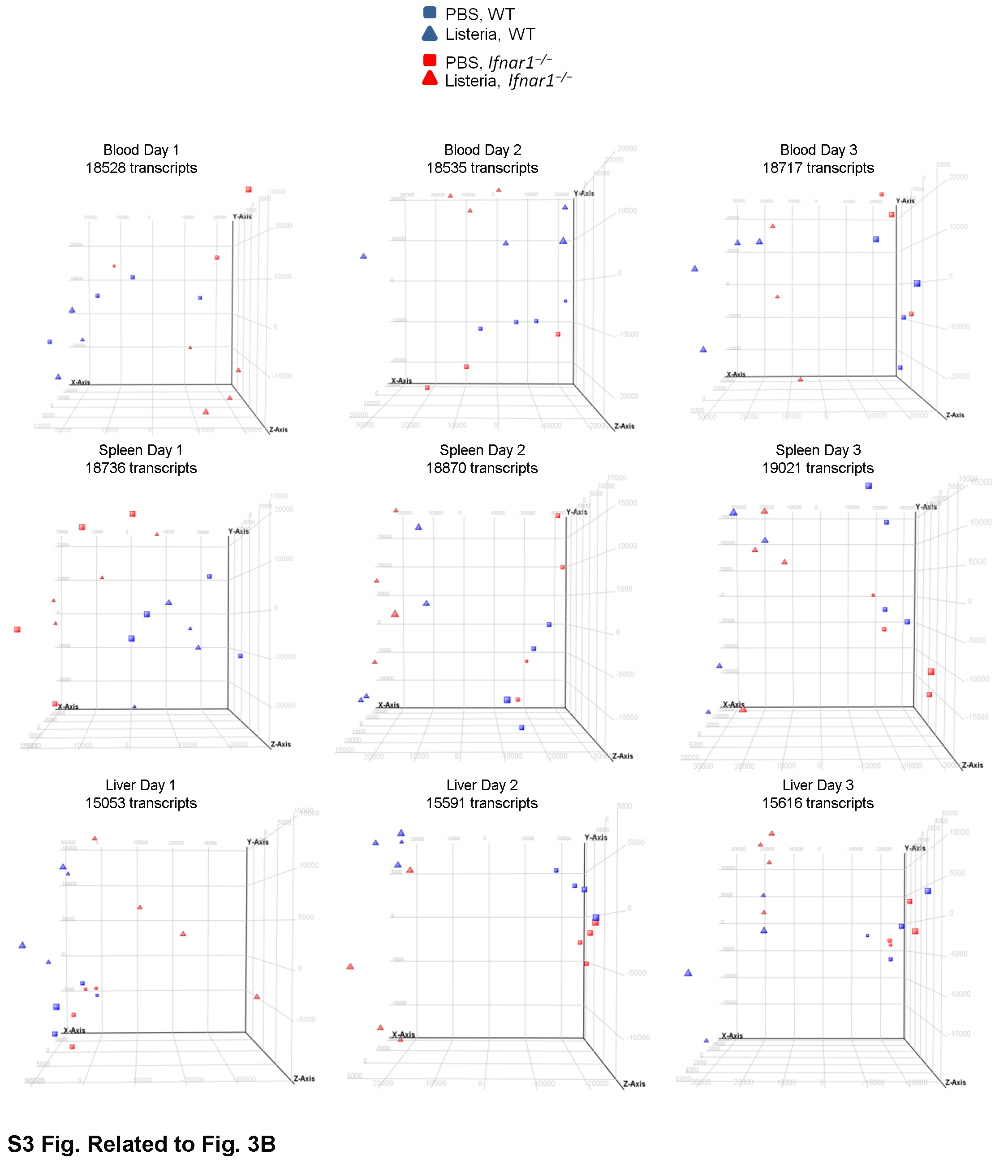

Supplement: S3 Fig — Principle component analysis of blood, spleen and liver transcripts passing QC filtering for uninfected and L. monocytogenes infected WT and Ifnar1-/- mice. Principal component analysis of transcripts significantly detected from background (p <0.01) separates infected from non-infected in blood, spleen and liver. (TIF) [file pone.0150251.s003.tif]

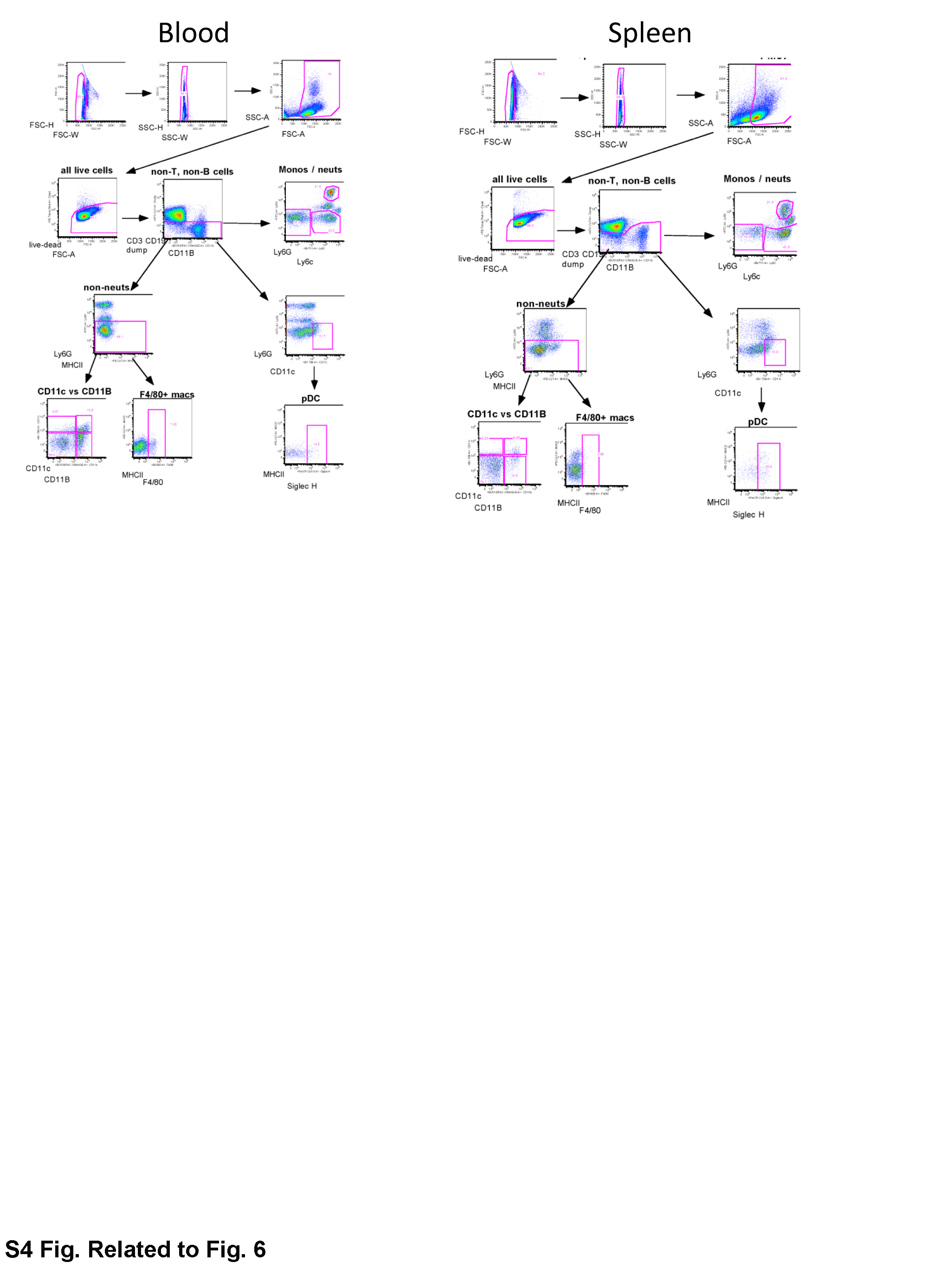

Supplement: S4 Fig — Flow cytometry to identify Ly6C+ monocytes and pDCs in blood and spleen. Representative images of the gating strategy to identify sub populations of myeloid cells from whole blood and spleen from L. monocytogenes infected mice. (TIF) [file pone.0150251.s004.tif]
