# Supplementary material for: Analysis of Transcriptional Signatures in Response to Listeria monocytogenes Infection Reveals Temporal Changes That Result from Type I Interferon Signaling
Source: PLoS One. 2016 Feb 26;11(2):e0150251. doi: 10.1371/journal.pone.0150251 (PMC4768944; doi:10.1371/journal.pone.0150251)
Supplement: S1 File — Top blood canonical pathways associated with transcripts that are differentially expressed in L. monocytogenes infected WT versus Ifnar1-/- mice relative to uninfected WT mice. Detailed gene heatmaps for all 30 IPA top pathways from Fig 4 except interferon signaling and antigen presentation. (PDF) [file pone.0150251.s005.pdf]

Activation of IRF by Cytosolic Pattern Recognition Receptors (16 of 50 pathway genes)

Symbol	WT infected / WT Ctrl			KO infected / WT ctrl			(WT infected / WT Ctrl) vs KO infected / WT ctrl		
	Day 1 154	Day 2 578 genes	Day 3 1343	Day 1 440	Day 2 684 genes	Day 3 1181	Day 1 84	Day 2 169 genes	Day 3 390
ADAR1									
c-Jun									
DAI									
IKBKE									
IL-10									
IRF-7									
IRF9									
ISG-54									
ISG15									
LGP2									
MAPK12									
MAPK9									
RIG-1									
STAT1									
STAT2									
TNFα									

Activation of IRF by Cytosolic Pattern Recognition Receptors (16 of 50 pathway genes)

Symbol	WT infected / WT Ctrl			KO infected / WT ctrl			(WT infected / WT Ctrl) vs KO infected / WT ctrl		
	Day 1 154	Day 2 578 genes	Day 3 1343	Day 1 440	Day 2 684 genes	Day 3 1181	Day 1 84	Day 2 169 genes	Day 3 390
ADAR1									
c-Jun									
DAI									
IKBKE									
IL-10									
IRF-7									
IRF9									
ISG-54									
ISG15									
LGP2									
MAPK12									
MAPK9									
RIG-1									
STAT1									
STAT2									
TNFα									

Altered T Cell and B Cell Signaling in Rheumatoid Arthritis (30 of 76 pathway genes)

Symbol	WT infected / WT Ctrl			KO infected / WT ctrl			(WT infected / WT Ctrl) vs KO infected / WT ctrl		
	Day 1 193	Day 2 792	Day 3 1956	Day 1 593	Day 2 930	Day 3 1728	Day 1 115	Day 2 228	Day 3 532
ACPA									
BAFFR									
CD79B									
CD86									
CXCL13									
FasL									
GM-CSF									
H2-Eb2									
HLA-DMA									
HLA-DMB									
HLA-DOA									
HLA-DOB									
IFN-γ									
IL-10									
IL-15									
IL-4									
IL12A									
IL18									
IL1B									
IL1RN									
M-CSF									
OPN									
TACI									
Tlr11									
Tlr13									
TLR2									
TLR4									
TLR6									
TLR7									
TNF									

Autoimmune Thyroid Disease Signaling (12 of 30 pathway genes)

Symbol	WT infected / WT Ctrl			KO infected / WT ctrl			(WT infected / WT Ctrl) vs KO infected / WT ctrl		
	Day 1 193	Day 2 792	Day 3 1956	Day 1 593	Day 2 930	Day 3 1728	Day 1 115	Day 2 228	Day 3 532
CD86									
FasL									
H2-Eb2									
HLA-A									
HLA-DMA									
HLA-DMB									
HLA-DOA									
HLA-DOB									
HLA-E									
IL-10									
IL-4									
Perforin									

Communication between Innate and Adaptive Immune Cells (28 of 61 pathway genes)

Symbol	WT infected / WT Ctrl			KO infected / WT ctrl			(WT infected / WT Ctrl) vs KO infected / WT ctrl		
	Day 1 193	Day 2 792	Day 3 1956	Day 1 593	Day 2 930	Day 3 1728	Day 1 115	Day 2 228	Day 3 532
BAFFR									
CCL3L3									
CCL4									
CCR7									
CD83									
CD86									
CD8B									
GM-CSF									
HLA-A									
HLA-E									
IFNγ									
IL-10									
IL-15									
IL-4									
IL12A									
IL18									
IL1B									
IL1RN									
IP-10									
RANTES									
TAC1									
Tlr11									
Tlr13									
TLR2									
TLR4									
TLR6									
TLR7									
TNF-α									

S1 File. Related to Fig. 4

Log Fold Change

-3 3

Communication between Innate and Adaptive Immune Cells (28 of 61 pathway genes)

Symbol	WT infected / WT Ctrl			KO infected / WT ctrl			(WT infected / WT Ctrl) vs KO infected / WT ctrl		
	Day 1 193	Day 2 792	Day 3 1956	Day 1 593	Day 2 930	Day 3 1728	Day 1 115	Day 2 228	Day 3 532
BAFFR									
CCL3L3									
CCL4									
CCR7									
CD83									
CD86									
CD8B									
GM-CSF									
HLA-A									
HLA-E									
IFNγ									
IL-10									
IL-15									
IL-4									
IL12A									
IL18									
IL1B									
IL1RN									
IP-10									
RANTES									
TAC1									
Tlr11									
Tlr13									
TLR2									
TLR4									
TLR6									
TLR7									
TNF-α									

Dendritic Cell Maturation (45 of 145 pathway genes)

Symbol	WT infected / WT Ctrl			KO infected / WT ctrl			(WT infected / WT Ctrl) vs KO infected / WT ctrl		
	Day 1 154	Day 2 578 genes	Day 3 1343	Day 1 440	Day 2 684 genes	Day 3 1181	Day 1 84	Day 2 169 genes	Day 3 390
CCR7									
CD1D									
CD83									
CD86									
DAP12									
FCGR1A									
FCGR2A									
FCGR2B									
FCGR3A/FCGR3B									
GM-CSF									
H2-Eb2									
HLA-A									
HLA-DMA									
HLA-DMB									
HLA-DOA									
HLA-DOB									
ICAM-1									
ICSBP									
IKBKE									
IL-10									
IL-15									
IL12A									
IL18									
IL1B									
IL1RN									
LTβR									
MAPK11									
MAPK12									
MAPK13									
MAPK9									
MYD88									
PDIA3									
PIK3CB									
PIK3CD									
PIK3R3									
PIK3R6									
PLCB2									
STAT1									
STAT2									
TLR2									
TLR4									
TNF-α									
TNFRSF1A									
TNFRSF1B									
TREM2									

Differential Regulation of Cytokine Production in Intestinal Epithelial Cells by IL-17A and IL-17F network (10 of 17 pathway genes)

Symbol	WT infected / WT Ctrl			KO infected / WT ctrl			(WT infected / WT Ctrl) vs KO infected / WT ctrl		
	Day 1 193	Day 2 792	Day 3 1956	Day 1 593	Day 2 930	Day 3 1728	Day 1 115	Day 2 228	Day 3 532
CCL2									
CCL4									
CCL5									
GM-CSF									
IFNγ									
IL-10									
IL-1β									
IL12A									
LCN2									
TNFα									

Glucocorticoid Receptor Signaling (40 of 254 pathway genes)

Symbol	WT infected / WT Ctrl			KO infected / WT ctrl			(WT infected / WT Ctrl) vs KO infected / WT ctrl		
	Day 1 193	Day 2 792	Day 3 1956	Day 1 593	Day 2 930	Day 3 1728	Day 1 115	Day 2 228	Day 3 532
ACTB									
BCL-2									
BCL-XL									
c-Fos									
CCL2									
CCL5									
CD163									
CD3E									
CSF2									
FCGR1									
HSP90AB1									
HSPA1A/HSPA1B									
HSPA5									
HSPA8									
ICAM1									
IFN-γ									
IKBKE									
IL-10									
IL-1β									
IL-4									
IL1R2									
IL1RA									
JAK3									
JUN									
Lipocortin									
MAPK11									
MAPK12									
MAPK13									
MAPK9									
MKP-1									
MRAS									
p21Cip1									
PIK3CB									
PIK3CD									
PIK3R3									
PIK3R6									
STAT1									
STAT3									
TNFα									
uPA									

Graft-versus-Host Disease Signaling (15 of 34 pathway genes)

Symbol	WT infected / WT Ctrl			KO infected / WT ctrl			(WT infected / WT Ctrl) vs KO infected / WT ctrl		
	Day 1 193	Day 2 792	Day 3 1956	Day 1 593	Day 2 930	Day 3 1728	Day 1 115	Day 2 228	Day 3 532
CD86									
FasL									
H2-Eb2									
HLA-A									
HLA-DMA									
HLA-DMB									
HLA-DOA									
HLA-DOB									
HLA-E									
IFNG									
IL18									
IL1B									
IL1RN									
Perforin									
TNF-α									

Granulocyte Adhesion and Diapedesis (37 of 147 pathway genes)

Symbol	WT infected / WT Ctrl			KO infected / WT ctrl			(WT infected / WT Ctrl) vs KO infected / WT ctrl		
	Day 1 193	Day 2 792	Day 3 1956	Day 1 593	Day 2 930	Day 3 1728	Day 1 115	Day 2 228	Day 3 532
CCL2									
CCL24									
CCL3L3									
CCL4									
CCL5									
CLDN15									
CXCL10									
CXCL13									
CXCL16									
Cxcl9									
CXCR2									
CXCR4									
FPR1									
FPR2									
GCSFR									
HRH2									
HSPB1									
ICAM1									
IL18									
IL18RAP									
IL1B									
IL1R2									
IL1RN									
ITGA2									
ITGAL									
MMP13									
MMP14									
MMP2									
MMP23B									
MMP25									
Ppbp									
SDC2									
SDF1									
TNF									
TNFRSF1A									
TNFRSF1B									
XCL1									

IL-10 Signaling (22 of 65 pathway genes)

Symbol	WT infected / WT Ctrl			KO infected / WT ctrl			(WT infected / WT Ctrl) vs KO infected / WT ctrl		
	Day 1 193	Day 2 792	Day 3 1956	Day 1 593	Day 2 930	Day 3 1728	Day 1 115	Day 2 228	Day 3 532
ARG2									
c-Fos									
c-Jun									
CCR5									
FCGR2A									
FCGR2B									
HMOX1									
IKBKE									
IL-10									
IL18									
IL18RAP									
IL1B									
IL1R2									
IL1RN									
LBP									
MAPK11									
MAPK12									
MAPK13									
SOCS3									
STAT3									
TNF									
TYK2									

IL-12 Signaling and Production in Macrophages (29 of 113 pathway genes)

Symbol	WT infected / WT Ctrl			KO infected / WT ctrl			(WT infected / WT Ctrl) vs KO infected / WT ctrl		
	Day 1 193	Day 2 792	Day 3 1956	Day 1 593	Day 2 930	Day 3 1728	Day 1 115	Day 2 228	Day 3 532
APOE									
c-FOS									
IFNγ									
IKBKE									
IL-10									
IL-12p35									
IL-18									
IL-4									
IL12RB1									
IRF-1									
IRF8									
JUN									
MAPK12									
MAPK13									
MAPK9									
MyD88									
p38β									
MAPK									
PIK3CB									
PIK3CD									
PIK3R3									
PIK3R6									
PU.1									
RAB7									
S100A8									
STAT1									
TLR2									
TLR4									
TNFα									

iNOS Signaling (15 of 43 pathway genes)

Symbol	WT infected / WT Ctrl			KO infected / WT ctrl			(WT infected / WT Ctrl) vs KO infected / WT ctrl		
	Day 1 193	Day 2 792	Day 3 1956	Day 1 593	Day 2 930	Day 3 1728	Day 1 115	Day 2 228	Day 3 532
CAMK4									
FOS									
IFNγ									
IKBKE									
IRF1									
JAK3									
JUN									
LBP									
MAPK11									
MAPK12									
MAPK13									
MyD88									
STAT1									
TLR4									
TYK2									

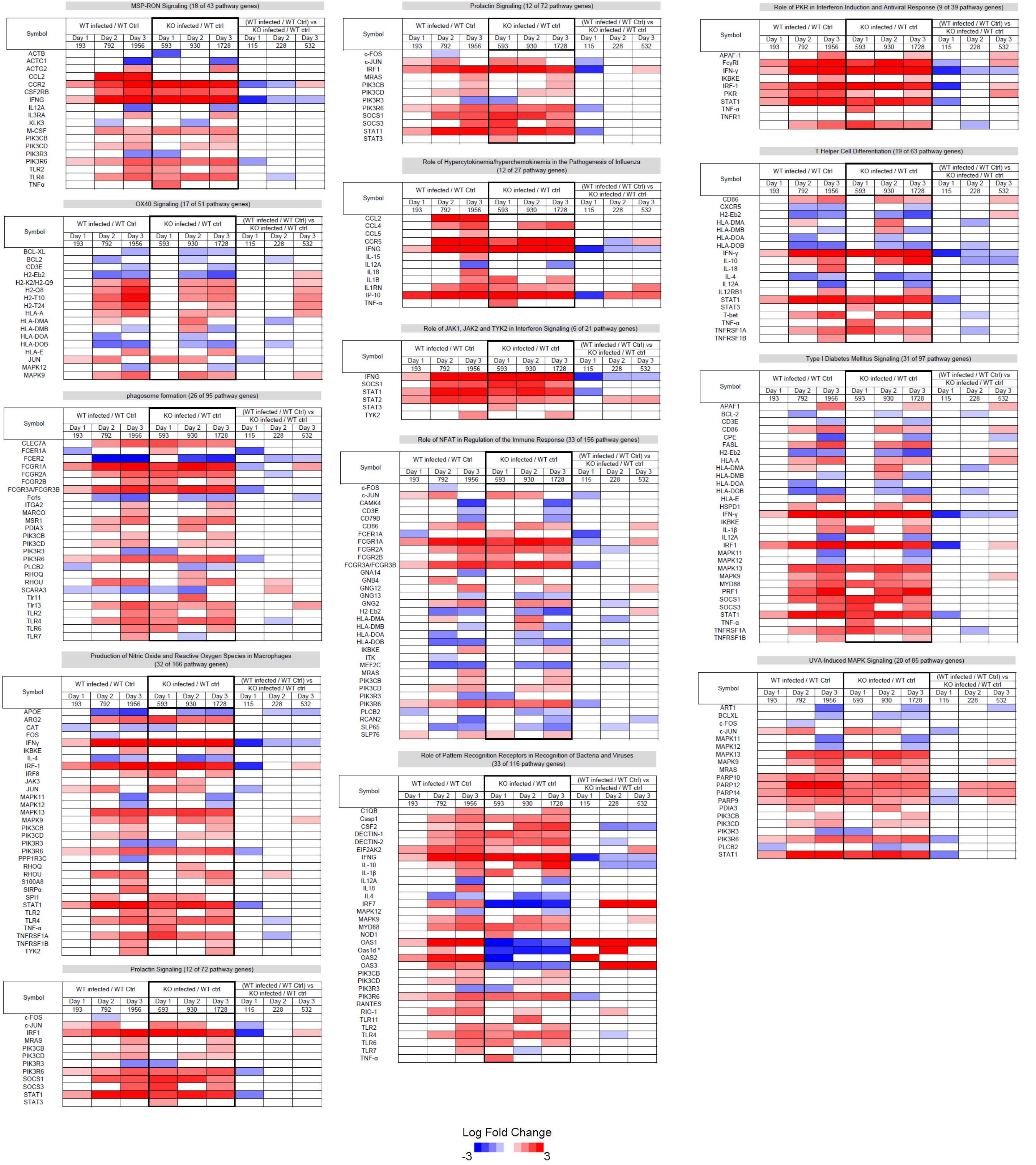
MSP-RON Signaling (18 of 43 pathway genes)
Symbol
WT infected / WT Ctrl
KO infected / WT ctrl
(WT infected / WT Ctrl) vs KO infected / WT ctrl
Day 1 Day 2 Day 3 Day 1 Day 2 Day 3 Day 1 Day 2 Day 3
193 792 1956 593 930 1728 115 228 532
ACTB ACTC1 ACTG2 CCL2 CCR2 CSF2RB IFNG IL12A IL3RA KLK3 M-CSF PIK3CB PIK3CD PIK3R3 PIK3R6 TLR2 TLR4 TNFα
OX40 Signaling (17 of 51 pathway genes)
BCL-XL BCL2 CD3E H2-Eb2 H2-K2/H2-Q9 H2-Q8 H2-T10 H2-T24 HLA-A HLA-DMA HLA-DMB HLA-DOA HLA-DOB HLA-E JUN MAPK12 MAPK9
phagosome formation (26 of 95 pathway genes)
CLEC7A FCER1A FCER2 FCGR1A FCGR2A FCGR2B FCGR3A/FCGR3B Fcrls ITGA2 MARCO MSR1 PDIA3 PIK3CB PIK3CD PIK3R3 PIK3R6 PLCB2 RHOQ RHOU SCARA3 Tlr11 Tlr13 TLR2 TLR4 TLR6 TLR7
Production of Nitric Oxide and Reactive Oxygen Species in Macrophages (32 of 166 pathway genes)
APOE ARG2 CAT FOS IFNγ IKBKE IL-4 IRF-1 IRF8 JAK3 JUN MAPK11 MAPK12 MAPK13 MAPK9 PIK3CB PIK3CD PIK3R3 PIK3R6 PPP1R3C RHOQ RHOU S100A8 SIRPα SPI1 STAT1 TLR2 TLR4 TNF-α TNFRSF1A TNFRSF1B TYK2
Prolactin Signaling (12 of 72 pathway genes)
c-FOS c-JUN IRF1 MRAS PIK3CB PIK3CD PIK3R3 PIK3R6 SOCS1 SOCS3 STAT1 STAT3
Prolactin Signaling (12 of 72 pathway genes)
c-FOS c-JUN IRF1 MRAS PIK3CB PIK3CD PIK3R3 PIK3R6 SOCS1 SOCS3 STAT1 STAT3
Role of Hypercytokinemia/hyperchemokinemia in the Pathogenesis of Influenza (12 of 27 pathway genes)
CCL2 CCL4 CCL5 CCR5 IFNG IL-15 IL12A IL18 IL1B IL1RN IP-10 TNF-α
Role of JAK1, JAK2 and TYK2 in Interferon Signaling (6 of 21 pathway genes)
IFNG SOCS1 STAT1 STAT2 STAT3 TYK2
Role of NFAT in Regulation of the Immune Response (33 of 156 pathway genes)
c-FOS c-JUN CAMK4 CD3E CD79B CD86 FCER1A FCGR1A FCGR2A FCGR2B FCGR3A/FCGR3B GNA14 GNB4 GNG12 GNG13 GNG2 H2-Eb2 HLA-DMA HLA-DMB HLA-DOA HLA-DOB IKBKE ITK MEF2C MRAS PIK3CB PIK3CD PIK3R3 PIK3R6 PLCB2 RCAN2 SLP65 SLP76
Role of Pattern Recognition Receptors in Recognition of Bacteria and Viruses (33 of 116 pathway genes)
C1QB Casp1 CSF2 DECTIN-1 DECTIN-2 EIF2AK2 IFNG IL-10 IL-1β IL12A IL18 IL4 IRF7 MAPK12 MAPK9 MYD88 NOD1 OAS1 Oas1d * OAS2 OAS3 PIK3CB PIK3CD PIK3R3 PIK3R6 RANTES RIG-1 TLR11 TLR2 TLR4 TLR6 TLR7 TNF-α
Role of PKR in Interferon Induction and Antiviral Response (9 of 39 pathway genes)
APAF-1 FcγRI IFN-γ IKBKE IRF-1 PKR STAT1 TNF-α TNFR1
T Helper Cell Differentiation (19 of 63 pathway genes)
CD86 CXCR5 H2-Eb2 HLA-DMA HLA-DMB HLA-DOA HLA-DOB IFN-γ IL-10 IL-18 IL-4 IL12A IL12RB1 STAT1 STAT3 T-bet TNF-α TNFRSF1A TNFRSF1B
Type I Diabetes Mellitus Signaling (31 of 97 pathway genes)
APAF1 BCL-2 CD3E CD86 CPE FASL H2-Eb2 HLA-A HLA-DMA HLA-DMB HLA-DOA HLA-DOB HLA-E HSPD1 IFN-γ IKBKE IL-1β IL12A IRF1 MAPK11 MAPK12 MAPK13 MAPK9 MYD88 PRF1 SOCS1 SOCS3 STAT1 TNF-α TNFRSF1A TNFRSF1B
UVA-Induced MAPK Signaling (20 of 85 pathway genes)
ART1 BCLXL c-FOS c-JUN MAPK11 MAPK12 MAPK13 MAPK9 MRAS PARP10 PARP12 PARP14 PARP9 PDIA3 PIK3CB PIK3CD PIK3R3 PIK3R6 PLCB2 STAT1
Log Fold Change
-3
3
