# Supplementary material for: Analysis of Transcriptional Signatures in Response to Listeria monocytogenes Infection Reveals Temporal Changes That Result from Type I Interferon Signaling
Source: PLoS One. 2016 Feb 26;11(2):e0150251. doi: 10.1371/journal.pone.0150251 (PMC4768944; doi:10.1371/journal.pone.0150251)
Supplement: S2 File — Top blood canonical pathways associated with transcripts that are differentially expressed in L. monocytogenes infected Ifnar1-/- versus WT mice relative to uninfected Ifnar1-/- mice. Detailed gene heatmaps for all 26 IPA top pathways from Fig 5 except interferon signaling and antigen presentation. (PDF) [file pone.0150251.s006.pdf]

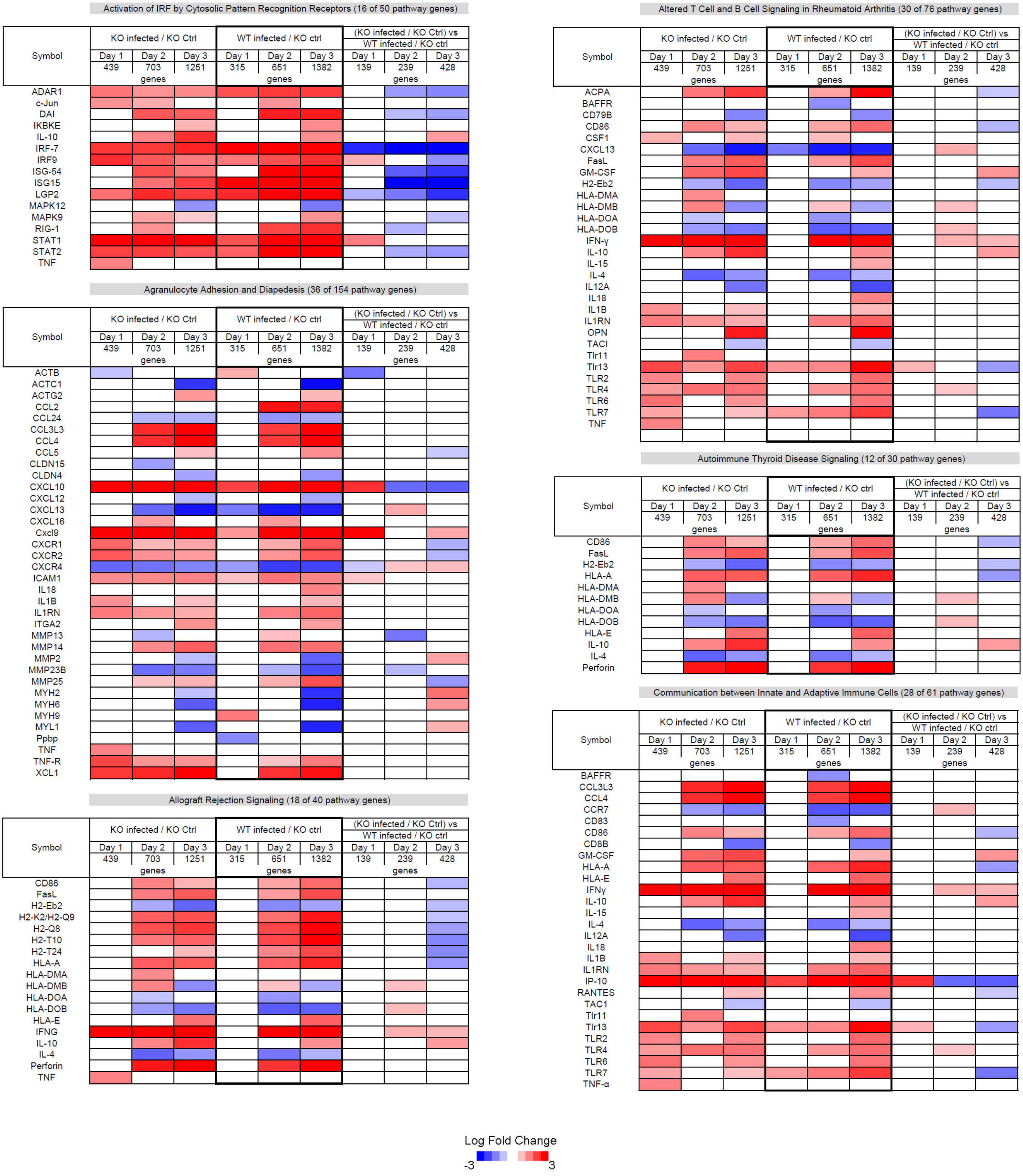

S2 File. Related to Fig. 5

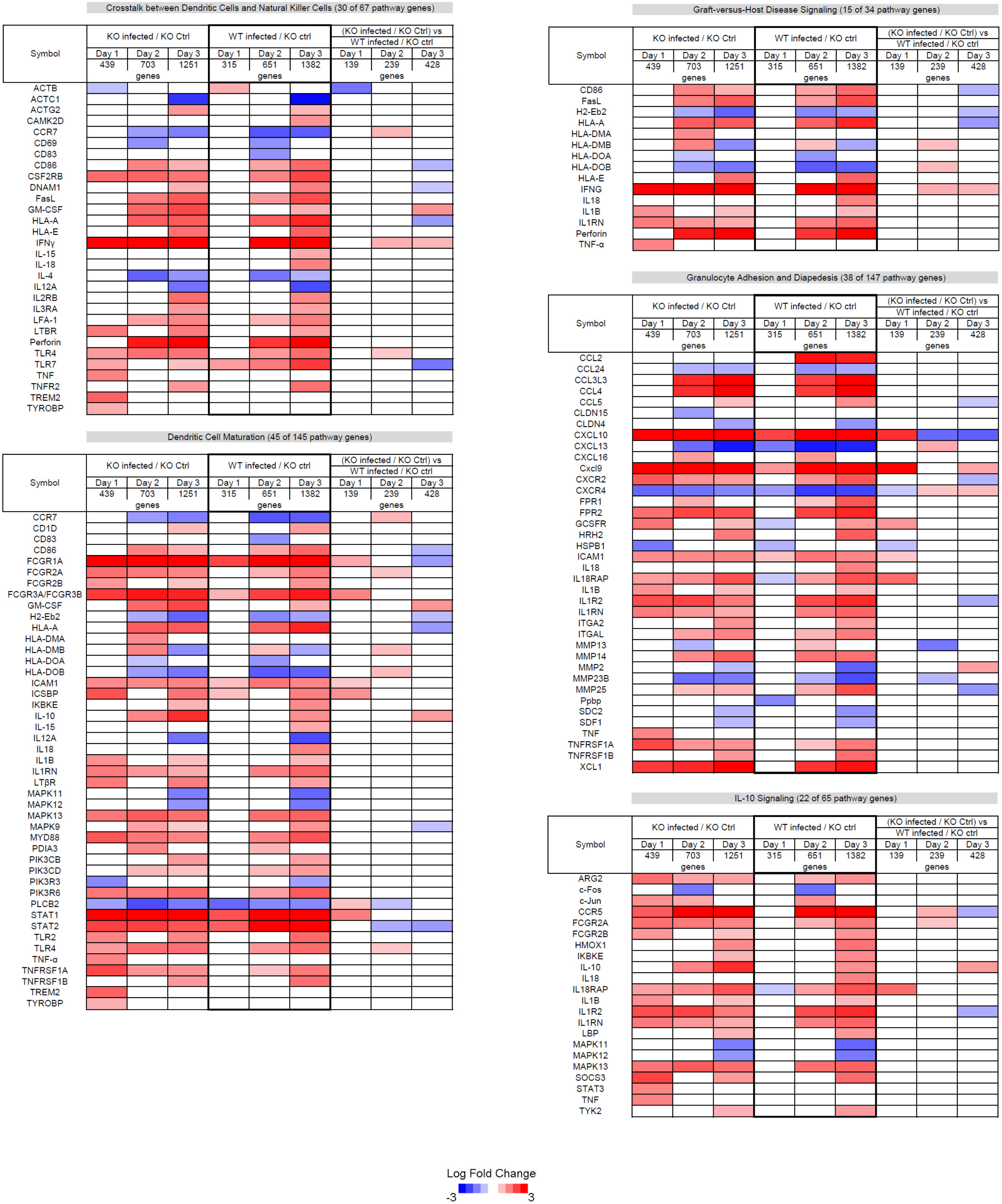
Crosstalk between Dendritic Cells and Natural Killer Cells (30 of 67 pathway genes)
Symbol
KO infected / KO Ctrl
WT infected / KO ctrl
(KO infected / KO Ctrl) vs WT infected / KO ctrl
Day 1 Day 2 Day 3
439 703 1251 genes
315 651 1382 genes
139 239 428 genes
ACTB
ACTC1
ACTG2
CAMK2D
CCR7
CD69
CD83
CD86
CSF2RB
DNAM1
FasL
GM-CSF
HLA-A
HLA-E
IFNγ
IL-15
IL-18
IL-4
IL12A
IL2RB
IL3RA
LFA-1
LTBR
Perforin
TLR4
TLR7
TNF
TNFR2
TREM2
TYROBP
Dendritic Cell Maturation (45 of 145 pathway genes)
CCR7
CD1D
CD83
CD86
FCGR1A
FCGR2A
FCGR2B
FCGR3A/FCGR3B
GM-CSF
H2-Eb2
HLA-A
HLA-DMA
HLA-DMB
HLA-DOA
HLA-DOB
ICAM1
ICSBP
IKBKE
IL-10
IL-15
IL12A
IL18
IL1B
IL1RN
LTβR
MAPK11
MAPK12
MAPK13
MAPK9
MYD88
PDIA3
PIK3CB
PIK3CD
PIK3R3
PIK3R6
PLCB2
STAT1
STAT2
TLR2
TLR4
TNF-α
TNFRSF1A
TNFRSF1B
TREM2
TYROBP
Graft-versus-Host Disease Signaling (15 of 34 pathway genes)
CD86
FasL
H2-Eb2
HLA-A
HLA-DMA
HLA-DMB
HLA-DOA
HLA-DOB
HLA-E
IFNG
IL18
IL1B
IL1RN
Perforin
TNF-α
Granulocyte Adhesion and Diapedesis (38 of 147 pathway genes)
CCL2
CCL24
CCL3L3
CCL4
CCL5
CLDN15
CLDN4
CXCL10
CXCL13
CXCL16
Cxcl9
CXCR2
CXCR4
FPR1
FPR2
GCSFR
HRH2
HSPB1
ICAM1
IL18
IL18RAP
IL1B
IL1R2
IL1RN
ITGA2
ITGAL
MMP13
MMP14
MMP2
MMP23B
MMP25
Ppbp
SDC2
SDF1
TNF
TNFRSF1A
TNFRSF1B
XCL1
IL-10 Signaling (22 of 65 pathway genes)
ARG2
c-Fos
c-Jun
CCR5
FCGR2A
FCGR2B
HMOX1
IKBKE
IL-10
IL18
IL18RAP
IL1B
IL1R2
IL1RN
LBP
MAPK11
MAPK12
MAPK13
SOCS3
STAT3
TNF
TYK2
Log Fold Change
-3
3

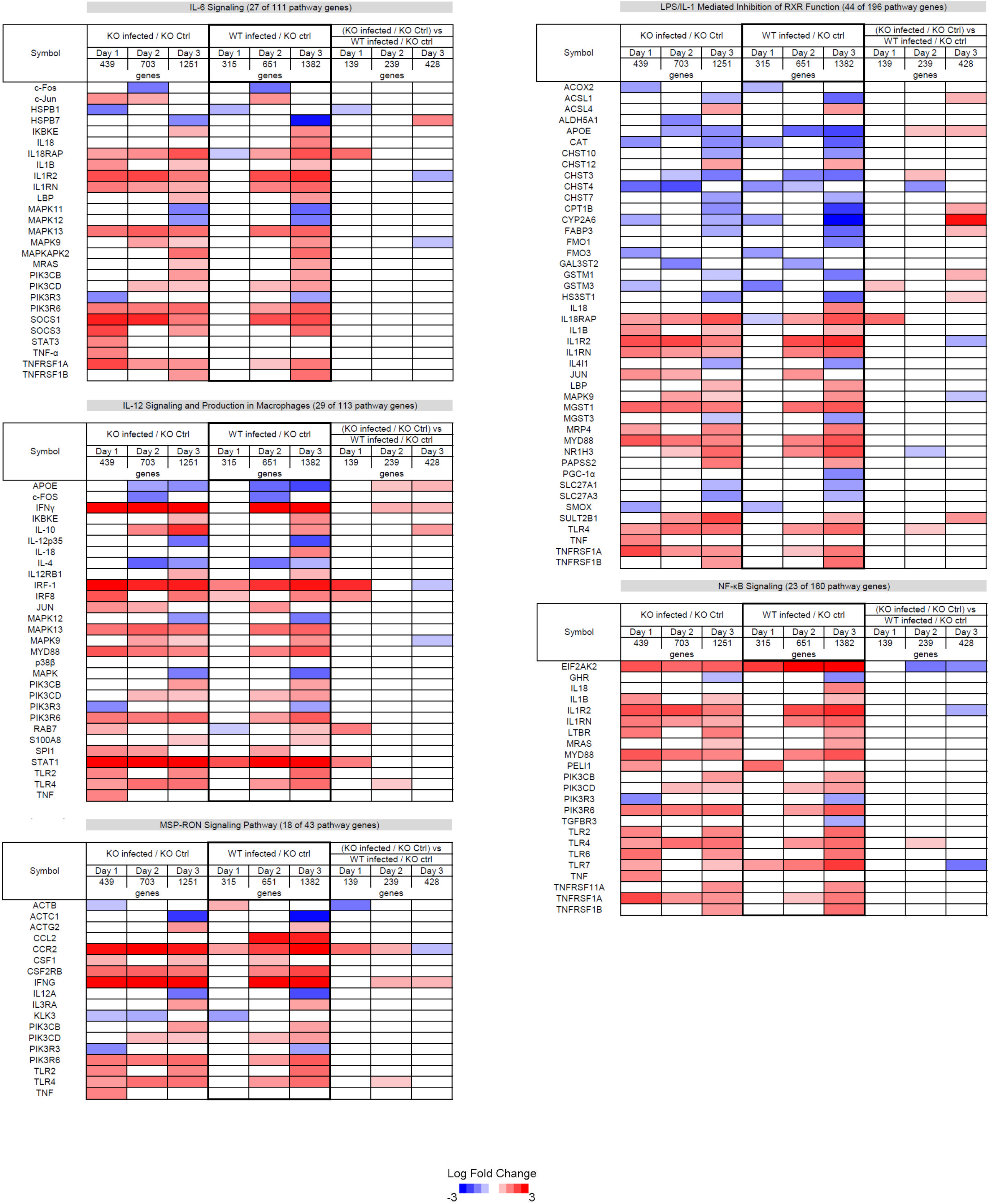
IL-6 Signaling (27 of 111 pathway genes)
Symbol
KO infected / KO Ctrl
WT infected / KO ctrl
(KO infected / KO Ctrl) vs WT infected / KO ctrl
Day 1 439
Day 2 703 genes
Day 3 1251
Day 1 315
Day 2 651 genes
Day 3 1382
Day 1 139
Day 2 239 genes
Day 3 428
c-Fos
c-Jun
HSPB1
HSPB7
IKBKE
IL18
IL18RAP
IL1B
IL1R2
IL1RN
LBP
MAPK11
MAPK12
MAPK13
MAPK9
MAPKAPK2
MRAS
PIK3CB
PIK3CD
PIK3R3
PIK3R6
SOCS1
SOCS3
STAT3
TNF-α
TNFRSF1A
TNFRSF1B
IL-12 Signaling and Production in Macrophages (29 of 113 pathway genes)
APOE
c-FOS
IFNγ
IKBKE
IL-10
IL-12p35
IL-18
IL-4
IL12RB1
IRF-1
IRF8
JUN
MAPK12
MAPK13
MAPK9
MYD88
p38β
MAPK
PIK3CB
PIK3CD
PIK3R3
PIK3R6
RAB7
S100A8
SPI1
STAT1
TLR2
TLR4
TNF
MSP-RON Signaling Pathway (18 of 43 pathway genes)
ACTB
ACTC1
ACTG2
CCL2
CCR2
CSF1
CSF2RB
IFNG
IL12A
IL3RA
KLK3
PIK3CB
PIK3CD
PIK3R3
PIK3R6
TLR2
TLR4
TNF
LPS/IL-1 Mediated Inhibition of RXR Function (44 of 196 pathway genes)
ACOX2
ACSL1
ACSL4
ALDH5A1
APOE
CAT
CHST10
CHST12
CHST3
CHST4
CHST7
CPT1B
CYP2A6
FABP3
FMO1
FMO3
GAL3ST2
GSTM1
GSTM3
HS3ST1
IL18
IL18RAP
IL1B
IL1R2
IL1RN
IL4I1
JUN
LBP
MAPK9
MGST1
MGST3
MRP4
MYD88
NR1H3
PAPSS2
PGC-1α
SLC27A1
SLC27A3
SMOX
SULT2B1
TLR4
TNF
TNFRSF1A
TNFRSF1B
NF-κB Signaling (23 of 160 pathway genes)
EIF2AK2
GHR
IL18
IL1B
IL1R2
IL1RN
LTBR
MRAS
MYD88
PELI1
PIK3CB
PIK3CD
PIK3R3
PIK3R6
TGFBR3
TLR2
TLR4
TLR6
TLR7
TNF
TNFRSF11A
TNFRSF1A
TNFRSF1B
Log Fold Change
-3
3

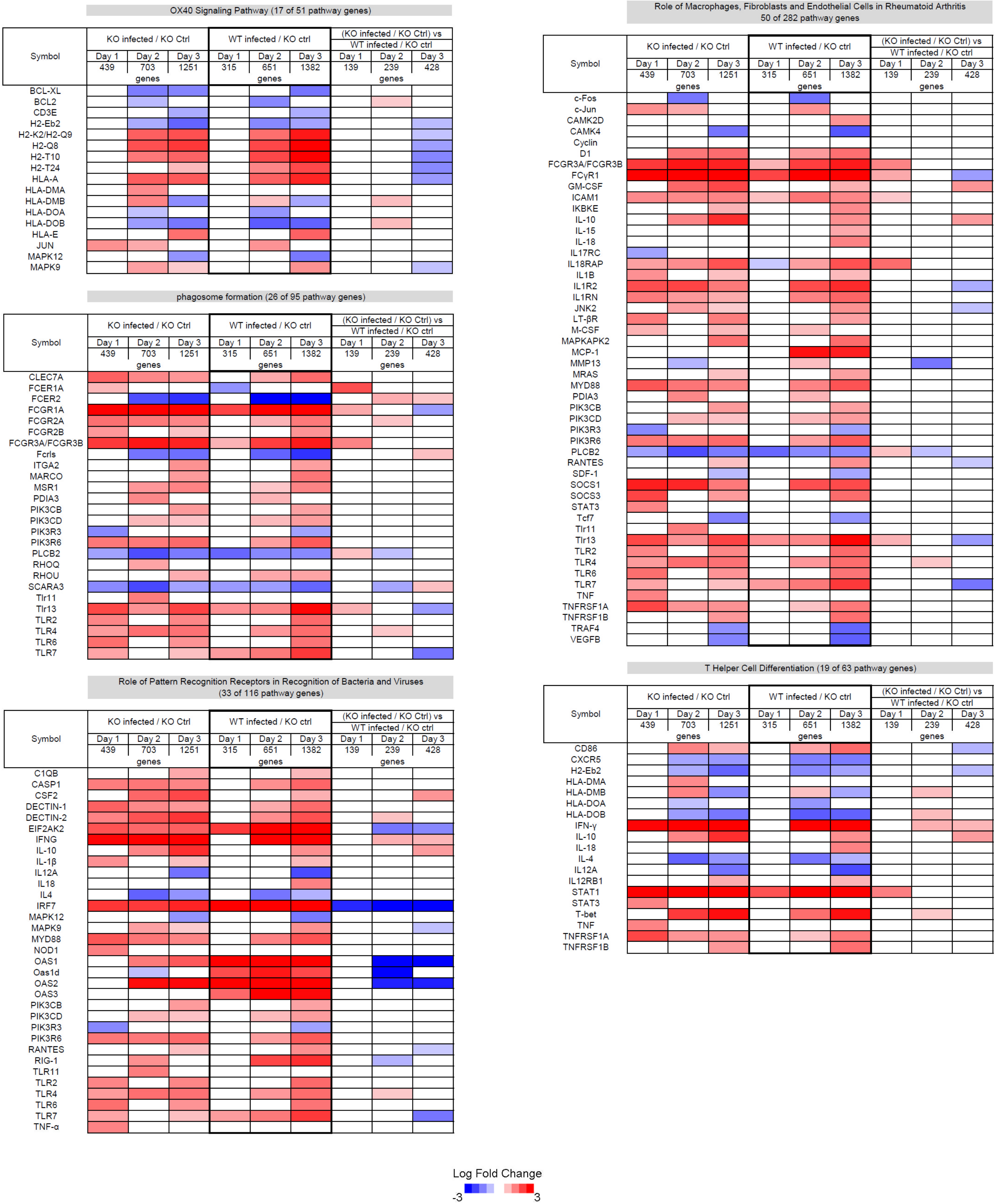
OX40 Signaling Pathway (17 of 51 pathway genes)
Symbol
KO infected / KO Ctrl
WT infected / KO ctrl
(KO infected / KO Ctrl) vs WT infected / KO ctrl
Day 1 Day 2 Day 3
439 703 1251 genes
315 651 1382 genes
139 239 428 genes
BCL-XL
BCL2
CD3E
H2-Eb2
H2-K2/H2-Q9
H2-Q8
H2-T10
H2-T24
HLA-A
HLA-DMA
HLA-DMB
HLA-DOA
HLA-DOB
HLA-E
JUN
MAPK12
MAPK9
phagosome formation (26 of 95 pathway genes)
CLEC7A
FCER1A
FCER2
FCGR1A
FCGR2A
FCGR2B
FCGR3A/FCGR3B
Fcrls
ITGA2
MARCO
MSR1
PDIA3
PIK3CB
PIK3CD
PIK3R3
PIK3R6
PLCB2
RHOQ
RHOU
SCARA3
Tlr11
Tlr13
TLR2
TLR4
TLR6
TLR7
Role of Pattern Recognition Receptors in Recognition of Bacteria and Viruses (33 of 116 pathway genes)
C1QB
CASP1
CSF2
DECTIN-1
DECTIN-2
EIF2AK2
IFNG
IL-10
IL-1β
IL12A
IL18
IL4
IRF7
MAPK12
MAPK9
MYD88
NOD1
OAS1
Oas1d
OAS2
OAS3
PIK3CB
PIK3CD
PIK3R3
PIK3R6
RANTES
RIG-1
TLR11
TLR2
TLR4
TLR6
TLR7
TNF-α
Role of Macrophages, Fibroblasts and Endothelial Cells in Rheumatoid Arthritis
50 of 282 pathway genes
c-Fos
c-Jun
CAMK2D
CAMK4
Cyclin D1
FCGR3A/FCGR3B
FCγR1
GM-CSF
ICAM1
IKBKE
IL-10
IL-15
IL-18
IL17RC
IL18RAP
IL1B
IL1R2
IL1RN
JNK2
LT-βR
M-CSF
MAPKAPK2
MCP-1
MMP13
MRAS
MYD88
PDIA3
PIK3CB
PIK3CD
PIK3R3
PIK3R6
PLCB2
RANTES
SDF-1
SOCS1
SOCS3
STAT3
Tcf7
Tlr11
Tlr13
TLR2
TLR4
TLR6
TLR7
TNF
TNFRSF1A
TNFRSF1B
TRAF4
VEGFB
T Helper Cell Differentiation (19 of 63 pathway genes)
CD86
CXCR5
H2-Eb2
HLA-DMA
HLA-DMB
HLA-DOA
HLA-DOB
IFN-γ
IL-10
IL-18
IL-4
IL12A
IL12RB1
STAT1
STAT3
T-bet
TNF
TNFRSF1A
TNFRSF1B
Log Fold Change
-3
3

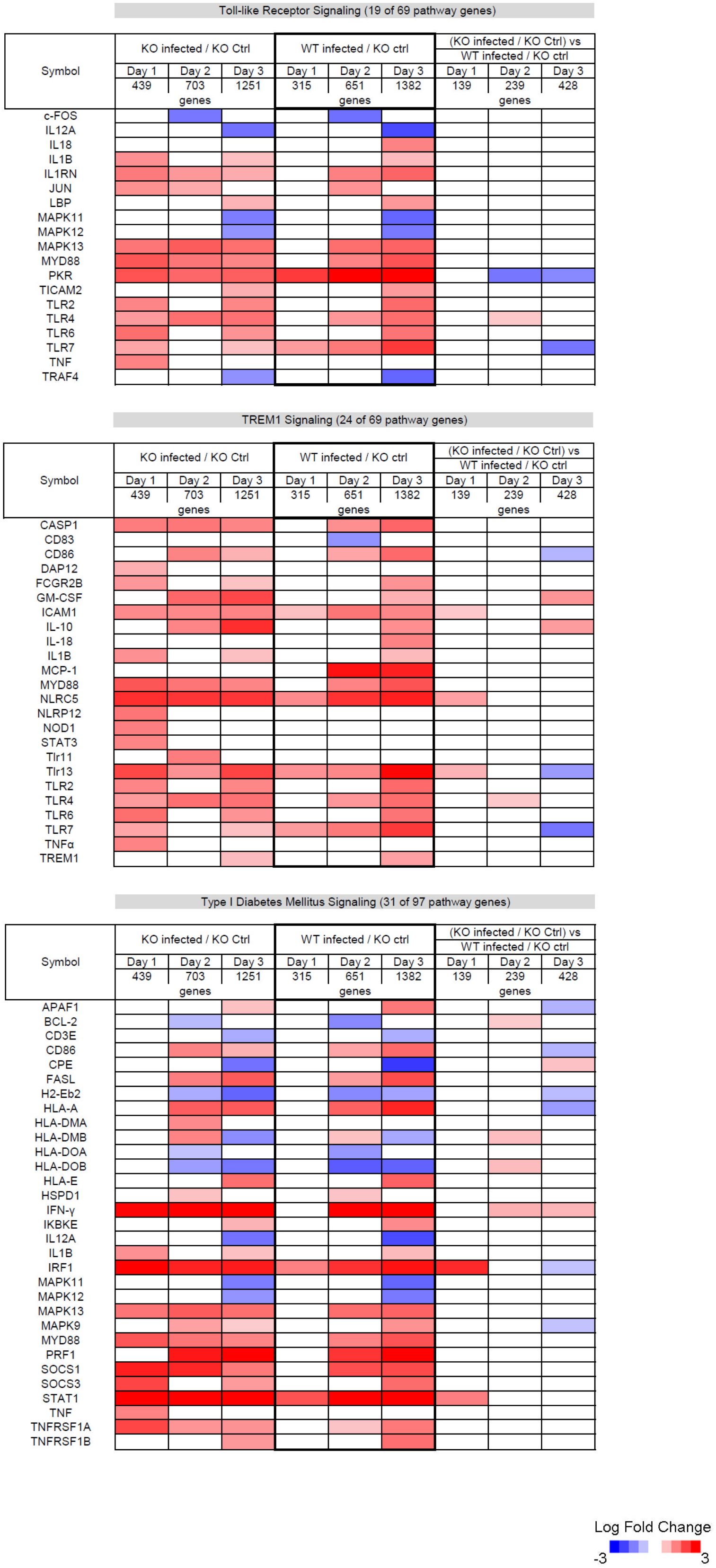
Toll-like Receptor Signaling (19 of 69 pathway genes)
Symbol
KO infected / KO Ctrl
WT infected / KO ctrl
(KO infected / KO Ctrl) vs WT infected / KO ctrl
Day 1 439
Day 2 703
Day 3 1251
genes
Day 1 315
Day 2 651
Day 3 1382
genes
Day 1 139
Day 2 239
Day 3 428
genes
c-FOS
IL12A
IL18
IL1B
IL1RN
JUN
LBP
MAPK11
MAPK12
MAPK13
MYD88
PKR
TICAM2
TLR2
TLR4
TLR6
TLR7
TNF
TRAF4
TREM1 Signaling (24 of 69 pathway genes)
Symbol
KO infected / KO Ctrl
WT infected / KO ctrl
(KO infected / KO Ctrl) vs WT infected / KO ctrl
Day 1 439
Day 2 703
Day 3 1251
genes
Day 1 315
Day 2 651
Day 3 1382
genes
Day 1 139
Day 2 239
Day 3 428
genes
CASP1
CD83
CD86
DAP12
FCGR2B
GM-CSF
ICAM1
IL-10
IL-18
IL1B
MCP-1
MYD88
NLRC5
NLRP12
NOD1
STAT3
Tlr11
Tlr13
TLR2
TLR4
TLR6
TLR7
TNFα
TREM1
Type I Diabetes Mellitus Signaling (31 of 97 pathway genes)
Symbol
KO infected / KO Ctrl
WT infected / KO ctrl
(KO infected / KO Ctrl) vs WT infected / KO ctrl
Day 1 439
Day 2 703
Day 3 1251
genes
Day 1 315
Day 2 651
Day 3 1382
genes
Day 1 139
Day 2 239
Day 3 428
genes
APAF1
BCL-2
CD3E
CD86
CPE
FASL
H2-Eb2
HLA-A
HLA-DMA
HLA-DMB
HLA-DOA
HLA-DOB
HLA-E
HSPD1
IFN-γ
IKBKE
IL12A
IL1B
IRF1
MAPK11
MAPK12
MAPK13
MAPK9
MYD88
PRF1
SOCS1
SOCS3
STAT1
TNF
TNFRSF1A
TNFRSF1B
Log Fold Change
-3
3
